# Shared “Core” Areas between the Pain and Other Task-Related Networks

**DOI:** 10.1371/journal.pone.0041929

**Published:** 2012-08-10

**Authors:** Franco Cauda, Diana M-E. Torta, Katiuscia Sacco, Elisabetta Geda, Federico D’Agata, Tommaso Costa, Sergio Duca, Giuliano Geminiani, Martina Amanzio

**Affiliations:** 1 CCS fMRI, Koelliker Hospital, Turin, Italy; 2 Department of Psychology, University of Turin, Turin, Italy; 3 Department of Neuroscience, AOU San Giovanni Battista, Turin, Italy; 4 Neuroscience Institute of Turin (NIT), University of Turin, Italy; National Research & Technology Council, Argentina

## Abstract

The idea of a ‘pain matrix’ specifically devoted to the processing of nociceptive inputs has been challenged. Alternative views now propose that the activity of the primary and secondary somatosensory cortices (SI, SII), the insula and cingulate cortex may be related to a basic defensive system through which significant potentially dangerous events for the body's integrity are detected. By reviewing the role of the SI, SII, the cingulate and the insular cortices in the perception of nociceptive and tactile stimuli, in attentional, emotional and reward tasks, and in interoception and memory, we found that all these task-related networks overlap in the dorsal anterior cingulate cortex, the anterior insula and the dorsal medial thalamus. A thorough analysis revealed that the ‘pain-related’ network shares important functional similarities with both somatomotor-somatosensory networks and emotional-interoceptive ones. We suggest that these shared areas constitute the central part of an adaptive control system involved in the processing and integration of salient information coming both from external and internal sources. These areas are activated in almost all fMRI tasks and have been indicated to play a pivotal role in switching between externally directed and internally directed brain networks.

## Introduction

Nociceptive stimuli activate a wide array of cortical areas including the primary (SI) and secondary (SII) somatosensory cortices [1], the insula, the cingulate cortex and the brainstem [2], [3], [4], [5]. How these activations relate to the complex experience of pain, that is, in normal conditions, the conscious perception triggered by nociceptive stimuli, remains debated. In recent years, pain research, in the quest of what constitues the ‘neural signature’ [6] of pain perception, has often intepreted the activation of the ‘pain matrix’ as representing the neural counterpart of the experience of pain. Although areas of greater or minor specificity for nociception have been proposed to form the ‘pain matrix’ [7], [8], [9], [10], [11], some interpretations of the ‘pain matrix’ diverge from what was initially proposed by Melzack with the idea of a ‘pain neuromatrix’ [12], see [13] for a more in depth discussion on the point). Indeed, in the original idea, supported by several past and recent findings, such activations were not defined as uniquely attributable to ‘pain’ as the very same areas could be activated also by non-nociceptive sensory stimuli [14], [15], [16], [17]. These results support the view that the activity in several areas forming the so called ‘pain matrix’ is far from constituting a uniquely faithul index of pain perception [1]. Indeed, in order to be ‘specific’ to a sensory stimulus or a task, activity in a brain region should *always* be evoked by that sensory stimulus and not by any other stimuli or tasks. In addition, specificity requires that the sensation be abolished when the brain region underpinning it is lesioned and that direct stimulation of that region evokes the sensation. If brain regions are active in response to a plurality of stimuli or tasks, it is more likely that those regions subserve functions common to all of the tasks.

From this perspective, it is of particular interest to study which activations are common to the ‘pain neuromatrix’ and to networks engaged in other cognitive/perceptual tasks [18].

Starting from the long-standing evidence that areas involved in the processing of painful stimuli can at least be involved in reward [19], emotional [15], [16], [17], [20], [21], mnesic [22], [23], [24] and attentional [16], [17], [21], [25] tasks as well as in the perception of tactile [14], [26], auditory and visual [15] stimulation, interoception [27] and action execution (motor) [28], we examined the current literature to investigate whether: i) all the networks recruited by these tasks share some common areas with the pain neuromatrix and, if such areas exist, ii) whether they share functional similarities. To answer the first question, we performed a PubAtlas search to explore whether the term ‘pain’ is more often employed in association with other terms in the scientific literature. For example, ‘pain’ and ‘emotion’ are two concepts frequently investigated together. Subsequently, we used the BrainMap database to retrieve areas of ‘term-related’ activations. In BrainMap, metadata are organized under three experiment-level fields: context, paradigm class and behavioral domain. We used the following query to limit the search to the corresponding category: Normal subjects AND (the term under study). We performed a voxel-based meta-analysis on the results of each of the separate searches. We studied the patterns of intersection among all these task-related networks using spatial probabilistic maps and conjunction analysis. In addition, we used the Meta-Analytic Connectivity Modeling (MACM) approach [29] to study the functional connectivity of the areas identified through conjunction analysis. This step allowed us to characterize the functional profile of possible areas of intersection. To answer the second question, namely, whether pain-related and non-pain-related networks share functional similarities, data were submitted to several data mining and network analysis techniques [30], [31]. These data analysis methods characterize how the networks are structured and connected to each other.

## Materials and Methods

### Selection of terms

The selection of the terms was limited to a series of keywords codified in the BrainMap [32], [33] database and identified as strongly related to pain as revealed by the PubAtlas search [34]. Indeed, as this study could have involved an extensive list of terms, we decided to confine the search by imposing a series of limitations. First, we excluded terms not codified as behavioral domains in the BrainMap database. Second, of those codified as behavioral domains in BrainMap, we only included the ones that were found to be strongly correlated with the term ‘pain’ as revealed by the Pub Atlas search.

### Phenotype Maps

We used Phenotype maps [35] to explore the current perception in the scientific literature of the interactions between the term ‘pain’ and other terms. To that end we used PubAtlas as a tool that makes it possible to explore the frequency with which terms are reported together in the scientific literature. PubAtlas [34] is a web-based application that supports examination and visualization of cognitive concepts published in PubMed. It attempts to provide “phenotype maps”, using a grid to map associations of large sets of terms. The strength of association is expressed as the natural logarithm of the Jaccard similarity index [36]. This index measures the similarity between sample sets, and is defined as the size of the intersection divided by the size of the union of the sample sets. Circular plots were generated using Circos (http://mkweb.bcgsc.ca/circos/).

### Literature meta-analysis

#### Selection of studies

Studies were retrieved using the BrainMap database [32], [33]. Separate systematic searches were conducted for studies performed between 1990 and 2010 involving painful stimulations and for studies involving ‘memory’, ‘touch’, ‘interoception’, ‘attention’, ‘action’, ‘emotion’ and ‘reward’. In BrainMap, metadata are organized under three experiment-level fields: context, paradigm class and behavioral domain. To limit the search to the corresponding category we used the following query: Normal subjects AND (the behavioural domain profile relative to the type of network to be examined). For example to extract the attentional network the search key was: “Normal subjects” AND Behavioural profile: “Attention”.

### Activation Likelihood Estimation (ALE)

We used an activation likelihood estimation (ALE) analysis to summarize the results of our database searches. Activation likelihood estimation (ALE) is a quantitative voxel-based meta-analysis method that can be used to estimate consistent activations across different imaging studies [37]. ALE maps of co-activations are derived on the basis of foci of interest, where multiple studies have reported statistically significant peak activation [37], [38].

### Conjunction analysis

We identified pivotal areas of task-related network intersections by performing a probabilistic superimposition and a conjunction analysis (100% probability of spatial overlap) of all eight task-related maps. To do so we first created a probabilistic map. At each spatial location, such maps represent the relative number of task-related networks leading to a significant task activity. After the creation of the map we applied a threshold to show only the voxels where 100% of the task-related networks are represented. Subsequent analyses were conducted on these regions of convergence.

### Meta-analytic connectivity modeling (MACM)

To analyze the meta-analytic connectivity (MACM) [39], [40] of the areas found as overlapping in the conjunction analysis, we queried BrainMap for papers reporting a co-activation of such areas. (e.g. reporting at least one focus in one of these areas). To calculate the MACM, the foci of papers reporting a focus in one of these areas were pooled using the ALE [41] algorithm. Each coordinate (focus) is modeled by a 3-D Gaussian distribution, defined by a full-width half-maximum (FWHM) of 10 mm. This width was based on previous work [42]. The ALE statistic was computed at every voxel in the brain. To assess the significance of the results, the values from the ALE images were tested against null distributions. An appropriate threshold was determined, while controlling the false discovery rate (FDR) at a significance level of p<0.05 [43].

### Representational similarity analysis

We transformed the ALE maps of task-related networks into a series of vectors containing all the values of the voxels of the original matrix [44], [45]. A representational similarity matrix was then constructed by calculating the correlation between each vector (r). The distance matrix was then constructed as 1-r.

### Multidimensional scaling

Multidimensional scaling (MDS) was applied to the distance matrix to provide a geometrical representation of the representational similarity results. In MDS, data to be analyzed are a collection of *I* objects on which a *distance function*, 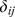 is defined. These distances are the entries of the *dissimilarity matrix* of the form:
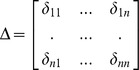
The goal of MDS is, given Δ, to find *N* vectors 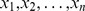 such that 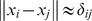 for all 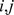. In classical MDS, this norm is the Euclidean distance. MDS attempts to find an embedding from the 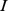 objects into 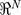 such that distances are preserved. Multidimensional scaling is a technique which finds a low-dimensional projection of points, where it tries to fit the given distances between points as well as possible. The shorter the Euclidean distance the greater the functional similarity.

### Similarity matrix reordering and clustering

The representational similarity matrix was reordered [46] to minimize the cross-correlation values off the diagonal and submitted to a hierarchical clustering algorithm. We employed hierarchical clustering to obtain a dendrogram of the task-related networks. Hierarchical clustering groups data over a variety of scales by creating a cluster tree. The tree is a multilevel hierarchy where clusters at one level are joined as clusters at the next level. This method allows the most appropriate level or scale to be chosen. The dendrogram was built using the Ward method which adopts an analysis of variance approach to evaluate the distances between clusters [47].

MDS, clustering and network analysis were performed using Orange Canvas (http://orange.biolab.si/doc/reference/) and visualized using Visual Understanding Environment (VUE; http://vue.tufts.edu/).

### Specificity for salience detection

An important question of this study was to ascertain whether our conjunction areas may be considered as specific for saliency detection. To address this issue, it was first necessary to specify which (and if) areas of the brain can be labeled as saliency-specific. A solution for this question can be obtained by combing meta-analytic tools and Bayesian inference techniques. We employed the Neurosynth Database [48] for this aim. Neurosynth is a highly automated brain mapping database and framework that, using text mining and meta-analytic procedures, can be used to explore the representational characteristics of several neural and cognitive states. This framework allows us to explore the specificity of an observed pattern of activation given a search term. The problem of specificity calls for the solution of both the forward and reverse inferences. The issue of the reverse inference [49] resides in the fact that the majority of neuroimaging studies provide a weak basis for determining what cognitive states a given brain pattern implies. Using Neurosynth we quantified the forward inference or the probability that there would be activation in specific brain regions given the presence of a particular term P(activation|term), and the reverse inference or the probability that a term would occur in an article given the presence of activation in a particular brain region P(term|activation).

## Results

**Figure 1:**
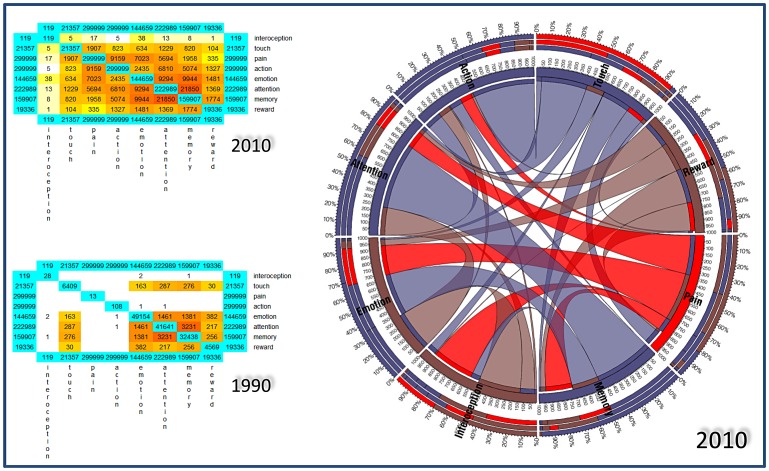
Phenotype maps. The left panel illustrates two heat maps of the co-occurrences of the terms ‘attention’, ‘emotion’, ‘touch’, ‘reward’, ‘interoception’, ‘memory’, ‘action’ and ‘pain’ thresholded to reveal only the strongest associations (natural log of Jaccard coefficient >−10) relative to the years 1990–2010. Warmer colors indicate a strong association between the two terms e.g. terms that were very frequently found together in an extensive literature search. The right panel shows a circular plot of the associations among the terms. Lexica are shown along the outside of the circles. The lines represent associations (Jaccard index) between lexica. The outer circles represent the association percentage explained by each lexica. Terms connected by a large strip are strongly associated e.g. were very frequently found together. Colors were arbitrarily assigned for visualization reasons. Circular plots were generated using Circos (http://mkweb.bcgsc.ca/circos/).

Eight terms were found to be more frequently related to ‘pain’ as revealed by the PubAtlas search and at the same time codified as categories in BrainMap. Such terms were: ‘memory’, ‘touch’, ‘interoception’, ‘attention’, ‘action’, ‘emotion’ and ‘reward’ (see Fig. 1).

As emerged from the results of the PubAtlas search, the term ‘pain’ has been cited with the other terms listed above with increasing frequency, suggesting a growing interest in the relationship between these concepts (see Fig. 1). Pain was found to have strong associations with all the other lexica but more frequently with attention, emotion and interoception.

**Figure 2:**
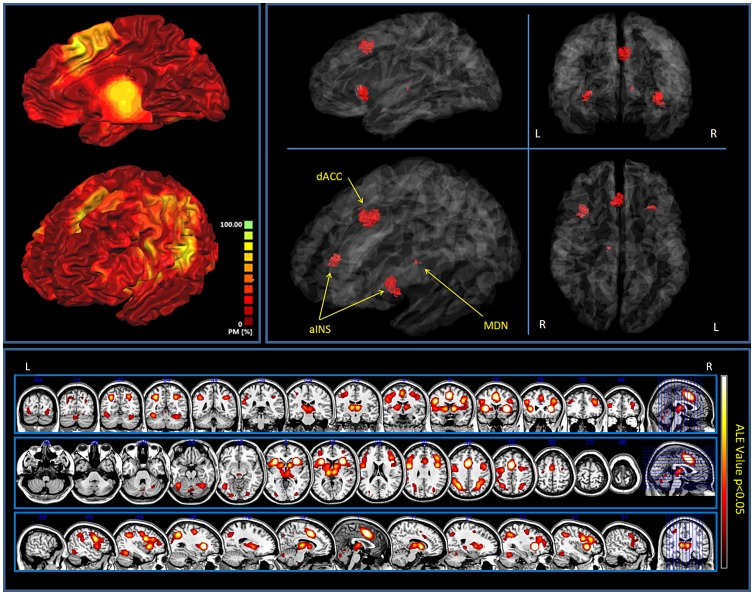
Areas of spatial overlap between Pain, Memory, Tactile stimulation, Interoception, Attention, Motor execution, Emotions and Reward networks. *Upper left panel*: The maps showed an increased probability of overlap between networks in the thalamus, anterior and mid-cingulate cortices, supplementary and pre-supplementary motor areas, sensorimotor, premotor, supramarginal and inferior parietal cortices. Areas with 0% probability of overlap are colored in red. A progressive increase in the probability of overlap is represented in shades of yellow. The probability map was calculated by summing the voxel value of each ALE-generated network and dividing this value by the number of networks (8). Single network maps created before the probability maps were thresholded at p<0.05, minimum cluster size k>100 mm^3^. *Upper right panel*: Conjunction analysis. We inspected pivotal areas of intersection between networks by performing a conjunction analysis of all eight maps (100% of spatial overlap). A comparison between this plot and the one presented in the middle panel of Figure 3 highlights the similarity of the results, although these were obtained with a different methodology (see text for further details). *Lower panel*: Meta-analytic connectivity of the network composed of the anterior insulae, the dorsal anterior cingulate cortex and the right medial dorsal thalamic nucleus. ALE maps were computed at an FDR-corrected threshold of p<0.05; minimum cluster dimension k>100 mm^3^ and visualized using Mricron (http://www.cabiatl.com/mricro/mricron/index.htm).

To seek areas of overlap among task-related networks, we first calculated their activation likelihood using the ALE method (see Figure S1). Afterwards, we calculated the spatial probability overlap among all ALE maps (see Fig. 2, upper left panel). The results of the conjunction analysis (see upper right panel in Fig. 2) showed that the eight task-related networks present a total overlap (100%) in three areas: the anterior insula, the dorsal anterior cingulate cortex and the right medial dorsal thalamic nucleus (see also Figure S1).

We examined the functional connectivity of these areas by performing a Meta-analytic Connectivity Modeling study. To this aim, we queried BrainMap for papers reporting a co-activation of all the regions of interest retrieved from the conjunction analysis. This analysis revealed a fronto-parietal group of areas including the anterior and dorsolateral prefrontal, dorsomedial superior frontal/anterior cingulate, inferior parietal lobule, and insular cortex (see Fig. 2, lower panel and also Figure S1 and Table S1). This network can also be detected using resting state functional connectivity techniques and was recently described as spatially interposed between the dorsal attentional network and the default mode network [50].

**Figure 3:**
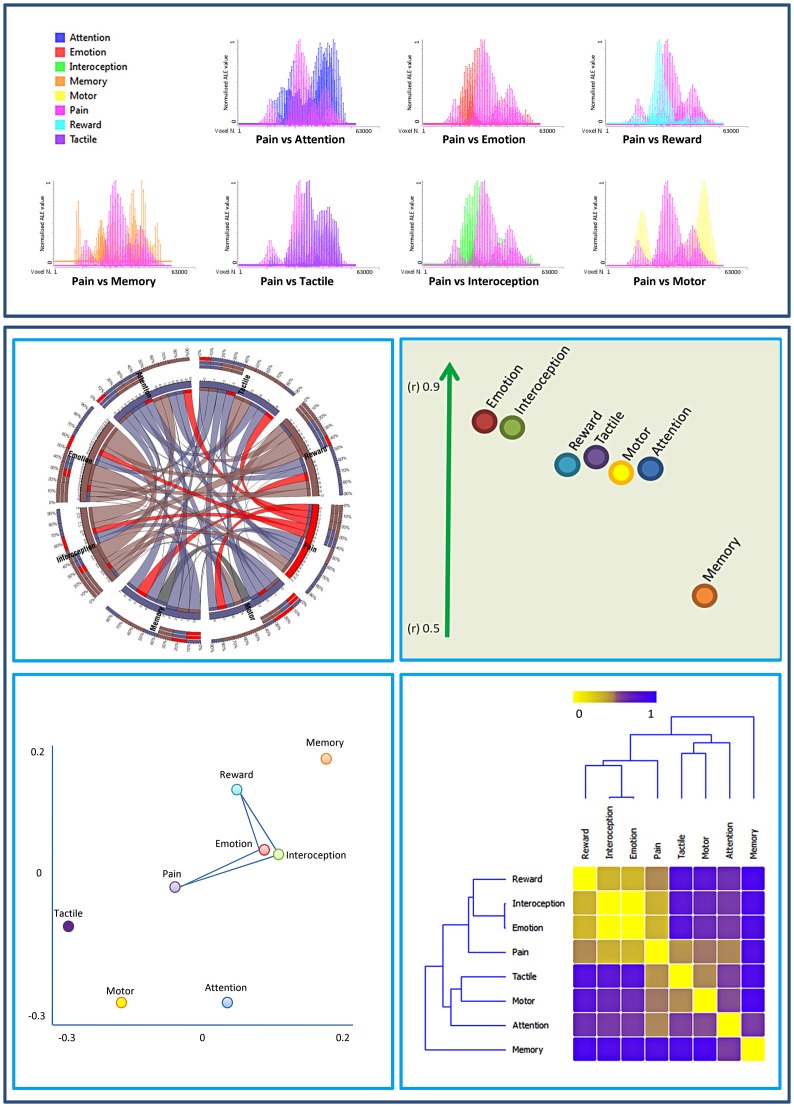
Network similarity. *Upper panel*: Comparison between the spatial profile of the ‘pain matrix’ and each of the other seven networks. Voxels are represented on the x-axis, normalized values of the ALE maps on the y-axis. *Middle right panel*: 1D Spatial similarity (expressed as spatial correlation) between the pain matrix and each of the other seven networks. The task-related networks are placed along the vertical axis on the basis of the spatial similarity with the pain matrix. The more a network is shifted towards the upper part of the graph the more its spatial pattern is similar to the spatial pattern of the pain network. *Middle left panel*: Circular plot showing the spatial similarity between networks. Similar networks are connected by a line. Network names are shown along the outside of the circles. The lines are intended to represent similarity (Spatial correlation) between networks. The width of the bands is proportional to the spatial similarity (expressed in percentage reported in the outer circle)explained by each network. Colors are chosen for representational purposes and have no statistical meanings, similarities with the pain network are coded in red. Circular plots were generated using Circos (http://mkweb.bcgsc.ca/circos/). *Lower right panel*: Distance matrix and hierarchical clustering of the eight networks. Networks with a low distance (distance = 1-spatial similarity) are placed close to each other. When a group of networks shows a high similarity it is grouped into a cluster sharing a similar spatial pattern. *Lower left panel*: Multidimensional scaling of the spatial profiles of the eight networks. Multidimensional scaling is a technique which finds a low-dimensional projection of points, where it tries to fit the given distances between points as well as possible. Points that are placed closely to each other have a similar spatial pattern. Points placed distantly from each other are characterized by a very different spatial pattern.

A representational similarity analysis [45] was performed to investigate the spatial similarity between task-related networks [45]. The spatial similarity/dissimilarity between pairs of networks can be quantified by computing a *distance matrix (the distance = 1- similarity)*. The results of the representational similarity analysis are shown in Fig. 3. Two clusters were identified (lower right panel): the first cluster including networks related to reward, interoception, emotion and pain. In this cluster interoception and emotion have a very similar spatial pattern, reflecting very similar functional properties. The second cluster was found to be composed of a more heterogeneous group where ‘detection of tactile stimuli’ and ‘action’ spatial patterns are more similar.

The spatial similarity analysis between the pain task-related network and each of the other networks (see Fig. 3, middle right panel) confirmed the clustering results showing that emotion and interoception have the highest spatial correlation with pain, followed by tactile, reward, attention and action.

The spatial profile and the circular plot (Fig. 3, upper and middle left panel) show that pain has an intermediate position between the two other clusters exerting connections with all task-related networks, in a very similar way as shown by the association between lexica in Figure 1. This is due to the fact that the network activated by the perception of painful stimuli is the one sharing more areas of overlap with the others as evidenced by the multidimensional scaling (Fig. 3 lower left panel). In accordance with these results, multidimensional scaling shows the pain network in a central position and the other networks in a more peripheral position.

**Figure 4:**
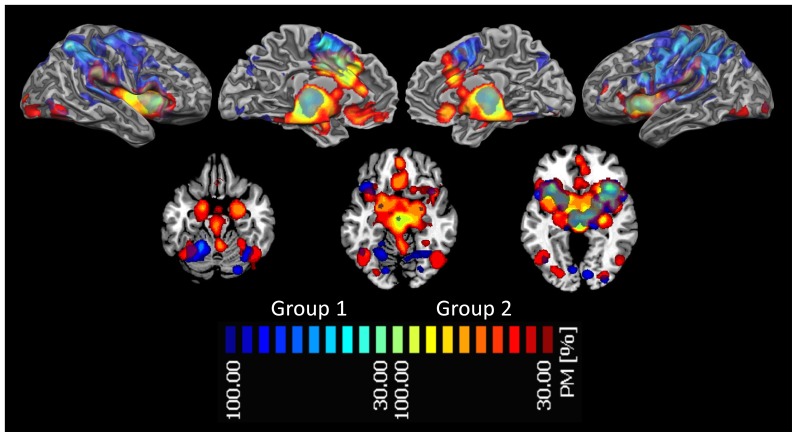
Brain Areas involved in the two groups of behavioral domains identified by the spatial similarity analysis. The maps show an increased probability of overlap between the behavioral domains identified by the spatial similarity analysis. Group 1 is constituted by ‘memory’, ‘touch’, ‘attention’ and ‘action’ (colors from green to blue represent an increased probability of overlap between the relative patterns of activations of the behavioral domains included in this group), group 2 by reward, interoception, emotion and pain action’ (colors from red to yellow represent an increased probability of overlap between the relative patterns of activations of the behavioral domains included in this group). The probability map was calculated by summing the voxel value of each ALE-generated network included in the group and dividing this value by the number of networks (8). Single network maps created before the probability maps were thresholded at p<0.05, minimum cluster size k>100 mm^3^.

Since the group composed of networks related to reward, interoception, emotion and pain showed a high similarity, we compared the structures involved in this cluster with the cluster composed of the other networks. Figure 4 shows the comparison of the probabilistic map of the two clusters. It is evident that the cluster composed of pain reward, interoception and emotion is characterized by a more pronounced insular, cingulate and subcortical profile including extensive thalamic, amygdalar and caudate activations whereas the other cluster shows a more sensorimotor, premotor, parietal and cerebellar pattern.

**Figure 5:**
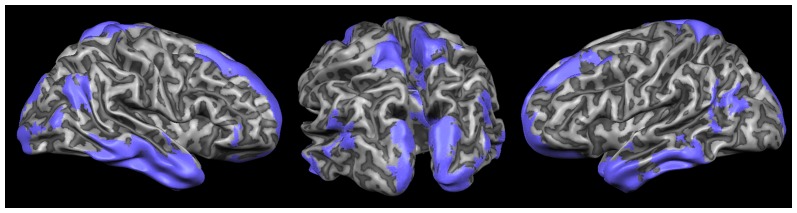
Areas showing a low percent of overlap between the eight task related networks. The figure shows the of the probabilistic map of the 20% of voxels showing the lower percent of overlap between the eight task related networks. ALE maps were computed at an FDR-corrected threshold of p<0.05; minimum cluster dimension k>100 mm^3^ and visualized using Mricron (http://www.cabiatl.com/mricro/mricron/index.htm).

Figure 5 shows the 20% of brain areas having the lesser overlap among networks, thus representing the structures that are more variable. These areas are prevalently in the precuneus, posterior parietal cortices, ventromedial and dorsomedial prefrontal cortices and visual areas.

### Speficity for salience detection

**Figure 6:**
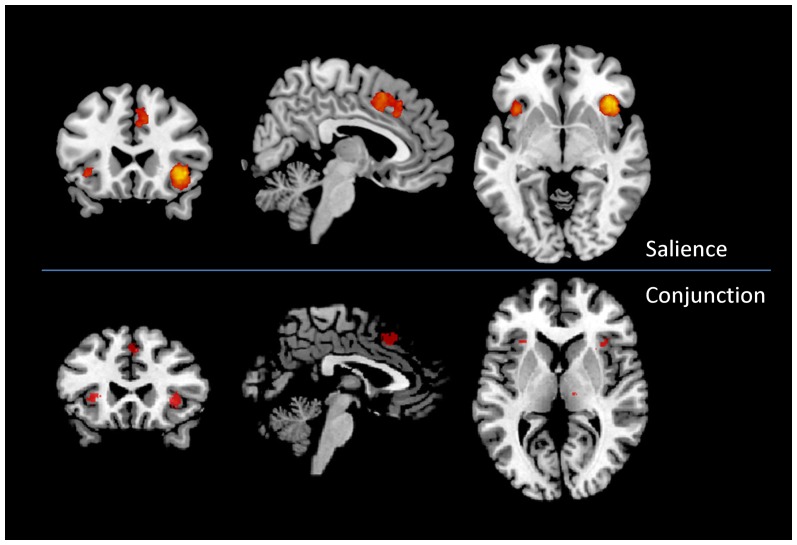
Comparison between the conjunction areas (voxels present in all the task related networks) and the areas specific for salience detection as retrived from Neurosynth. Upper panel: Neurosynth map, values from red to yellow represent increasing probabilities in a forward inference Bayesian model. Lower panel: Conjunction analysis between all task related networks. ALE maps were computed at an FDR-corrected threshold of p<0.05; minimum cluster dimension k>100 mm^3^ and visualized using Mricron (http://www.cabiatl.com/mricro/mricron/index.htm).

When examining with the Neurosynth the specificity of the brain response to salience detection (Fig. 6), we found an almost total coincidence between our “conjunction” areas and the salience detection areas.

## Discussion

In the present study, we started from the evidence that areas involved in the elaboration of painful stimuli are also recruited to process other sensory stimuli [15] and cognitive and motor tasks [14], [19], [20], [22], [25], [26], [27], [51] and we investigated: i) the existence of common brain regions that are active across a group of task-generated networks (including the network devoted to the processing of painful stimuli), and ii) whether these networks share any functional similarities.

By applying voxel-based meta-analysis, probabilistic and conjunction analysis to the results of a BrainMap query we observed that three areas are active parts of all the eight selected task-related networks: the anterior insula, the dorsal anterior cingulate cortex and a small portion of the right dorsal medial thalamus. The present findings indicate that a wide range of task- related networks present activations that *per se* are highly unspecific to the task at hand. What is more, these three areas present a fronto-parietal pattern of functional connectivity typical of the ‘fronto parietal control network’ [50], a brain network that has been proposed to be in charge of integrating information from the dorsal attentional system (externally directed) and the default mode network (internally directed). It has been demonstrated that these areas activate, rather than in a task-specific manner, in relation to a degree of personal salience, when homeostatic, cognitive or emotional stimuli require changes in the sympathetic system response. For instance, the medial dorsal thalamus (one of the three areas of overlap) is involved in several functions related to attention and salience processing and is functionally and anatomically connected with the anterior cingulate cortex, the dorsolateral prefrontal cortex and the insula [52], [53].

According to some authors, the fronto-parietal network, identified also in resting state conditions [50], consists of a set of frontal and parietal regions, which identify the most relevant stimuli in the environment and integrate them with visceral, autonomic, and hedonic markers [54], thus playing a crucial role in the integration of internal and external information. In this view, the activation of the insula, dorsal anterior cingulate cortex and the thalamus in all task-related networks suggests a common functional significance; indeed, these regions may be hubs devoted to the exchange of information between internal and external sources. As hubs, they allow the integration of afferent homeostatic, environmental, hedonic, motivational, social and cognitive activity [27], [55], [56]. In addition, they facilitate task-related information processing by initiating appropriate transient control signals focusing attention on external salient stimuli [57]. Converging evidence from a number of brain imaging studies across several task domains has suggested that the anterior insula and the anterior cingulate cortex are activated whenever an exogenous sensory stimulus is considered as salient or an endogenous perceptual task is challenging [58]. In the model proposed by Craig [27], [55], [56] the insula integrates salient activities. According to this author, starting from its most posterior parts and moving towards its anterior ones, the insula gradually receives and integrates afferent information to produce a ‘global emotional moment’, which represents the sentient self at one moment in time [27], [55], [56]. Furthermore, Menon and Uddin [57] recently proposed that the function of the anterior insula is that of facilitating task-related information processing by initiating appropriate transient control signals focusing attention on external stimuli. Together with the cingulate cortex, the insula would respond to a degree of subjective salience, integrating the most relevant internal and external elements with the ultimate aim of guiding behavior [41], [59], [60]. Indeed the anterior insula plays a critical and causal role in activating the control network and deactivating the default mode network [57], [61]. Its activity is then followed by that of the anterior cingulate cortex. This would suggest that the right anterior insula participates in the coordination of task performance across behavioral tasks with different perceptual and response demands [59]. Accordingly, it has been hypothesized that the anterior insula provides a link between attention-related problem-solving and salience systems during the coordination and evaluation of task performance [57], [61], [62].

Taken together, these previous findings and our present results support the view that the anterior insula and the dorsal anterior cingulate cortex may constitute crucial hubs of a multimodal network involved in the detection of salient events for the body which also integrate homeostatic information coming from the internal source.

To further substantiate this hypothesis we explored, with the use of the Neurosynth database, which brain areas present a greater specificity for saliency detection. The results indicated a higher specificity for saliency detection in the dorsal anterior cingulate cortex and the insula, thus confirming our proposal. Areas of minor overlap were those often deactivated in fMRI tasks. In addition, our results highlighted the absence of areas selectively activated by saliency detection, thus suggesting that this requirement is common to all fMRI tasks.

Importantly, we do not exclude the possibility that a task-specific activity exists; this may be reflected in the pattern of functional connectivity that is established for every task [63]. In addition, it should be noted that our conclusions are mainly based on the spatial overlapping of the networks. However, from these analyses we cannot extrapolate much information regarding the timing of activation of the areas. For instance, early activation of the thalamus reflects mainly afferent data transmission, but thalamic activations may also reflect, when occurring later, cortico-thalamic modulatory activity.

Network analysis was performed to investigate whether the eight task-related networks share any functional similarities. Graph analysis (see the dendrogram in Figure 3 lower right panel) evidenced two clusters: an affective/vegetative group comprising pain, emotion, interoception and reward and a sensorimotor/attentional group including touch, motor and attention, each with a specific pattern of activations (Fig. 4): the first cluster with a more insular, cingulate and subcortical profile includes thalamic, amygdalar and caudate activations whereas the second cluster shows a more sensorimotor, premotor, parietal and cerebellar pattern of activations.

Since all the task-related networks share a common group of brain areas this classification specifically highlights the differences between networks. Interestingly in this analysis the touch and the pain networks belong to two different clusters. Moreover, multidimensional scaling (see Fig. 3 lower left panel) and the analysis of the patterns of activations of the two clusters of tasks (Fig. 4), revealed that the pain-related network has a pivotal, central position between the somatosensory and somatomotor group and the emotional-interoceptive one, suggesting that it is the one that shares more connections with other task-related networks. This is in line with the idea of the evolutionary function of pain, which is aimed at signaling a potential threat or damage to the individual in order to motivate escape [1], [64]. For this ultimate purpose of survival, the brain has to integrate a great deal of information, coming from both the external and internal environments (see Figure S1). This would explain why brain areas related to the processing of pain have to be so functionally connected with other networks. These results appear to suggest that the pain-related network, in addition to reflecting the commonly distributed stimulus-triggered attention-related responses, also reflects affective and/or vegetative responses. Indeed, recent models, besides reconsidering a part of the activity of the so called pain matrix as related to mechanisms of attentional trigger [1], [64] have also underlined the importance of top-down factors, such as motivation and goals in the perception of painful stimuli [65].

Our analysis is focused on areas that are common to all task-related networks but it can be argued that since the brain can recognize and react to a series of different stimuli and tasks, for the comprehension of the detection system also the structures that have a high variability between tasks are important. Interestingly, most of these areas (Fig. 5) like precuneus posterior parietal, ventromedial, dorsomedial prefrontal cortices and visual areas are part of the group of areas that are often deactivated by common active fMRI tasks called “task-induced deactivations” [66]. A possible explanation stems from the observation that the magnitude of task-induced deactivation is strongly linked to the cognitive demands of tasks and thus is greatly variable in relation to different external requirements [67], [68].

A shortcoming of this study is that we chose to limit the number of terms (and consequently the number of task-related networks) to eight. The decision to use a database search forced us to limit the number of terms (and consequently of task-related networks) to the eight previously described. For this reason, we cannot exclude the possibility that other networks, for example those related to other sensory modalities such as visual or auditory processing, may demonstrate a similar involvement as the ones employed in this analysis. In addition, as we only selected terms codified as behavioral domain classes in the BrainMap database, we could not explore terms such as ‘nociceptive’, ‘haptics’ ‘allodynia’ or differential activations related to ‘top-down’ or ‘bottom up’ attention, as well as to ‘short-term’ and ‘long-term’ memory. The inclusion of such additional terms and subdivisions might have added a more fine grained picture of how other networks relate to the pain network.

## Conclusions

We have shown that when investigating the relationship among areas of the ‘pain matrix’, different functional task-generated networks and their areas of overlap, a ‘core system’ constituted by three areas emerges (the insula, dorsal anterior cingulate cortex and thalamus), the activity of this system may be considered as integrative of salient stimuli coming from the *external* and *internal* world. This finding may support the hypothesis that areas of the ‘pain matrix’ represent not only activation of a system for detecting salient stimuli, but also that components of such network are intimately linked to the updating of internal states of the individual and to aims and goals. These components inevitability interplay with the perception and processing of external potentially dangerous stimuli. We therefore propose that a common network exists that might constitute not only a saliency detection system for the body, but also a control system for survival.

## Supporting Information

Figure S1 **Additional figures and infographics.**
(PDF)

Table S1 **Additional tables showing the papers involved in the study and the results of each topic-related metaanalysis.**
(PDF)
