# Supplementary material for: Shared “Core” Areas between the Pain and Other Task-Related Networks
Source: PLoS One. 2012 Aug 10;7(8):e41929. doi: 10.1371/journal.pone.0041929 (PMC3416807; doi:10.1371/journal.pone.0041929)
Supplement: Figure S1 — Additional figures and infographics. (PDF) [file pone.0041929.s001.pdf]

SUPPLEMENTARY FIGURES

FIG S1 TASK-RELATED NETWORKS

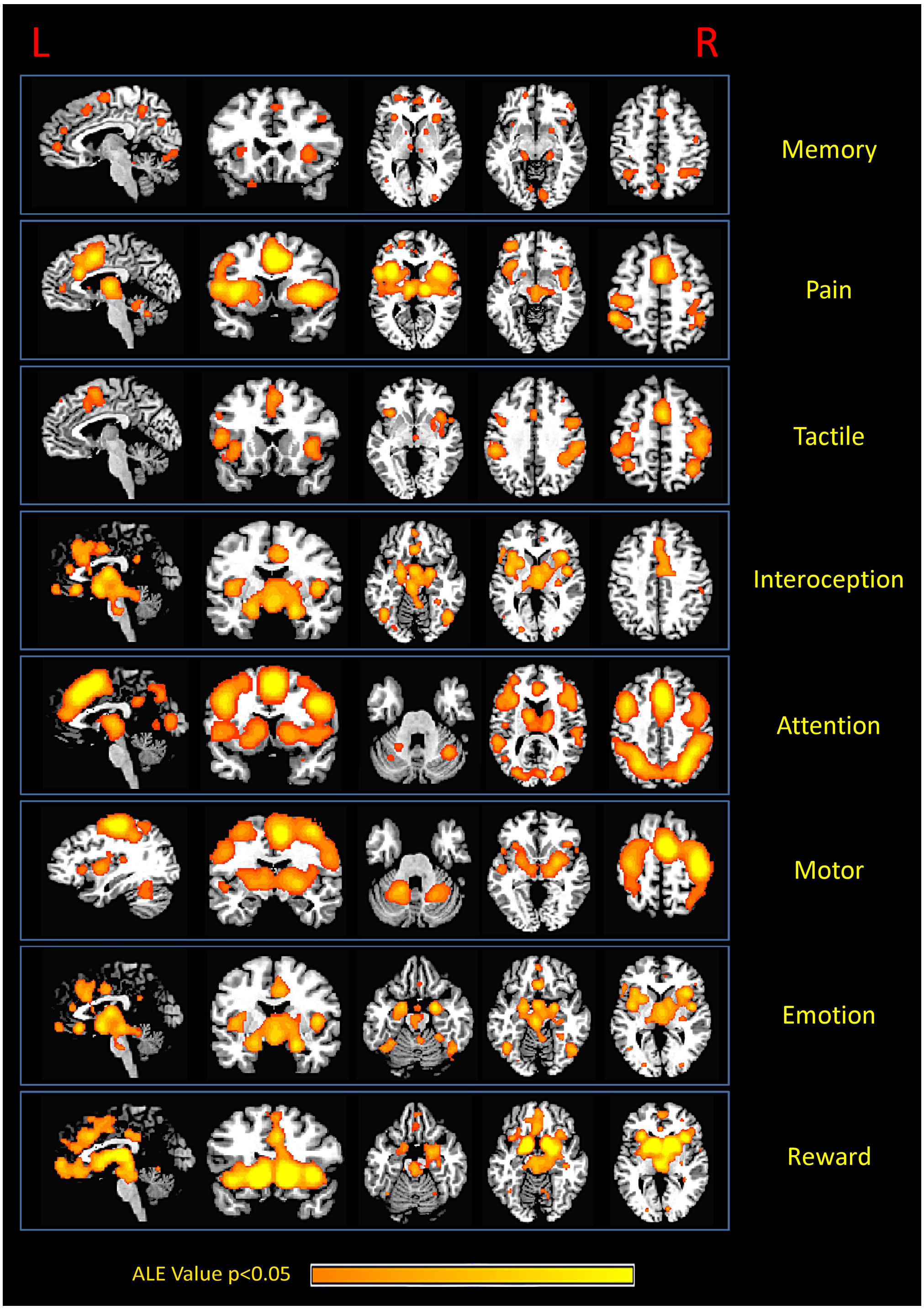

ACTIVATION LIKELIHOOD ESTIMATION STATISTICAL MAPS RELATIVE TO THE ACTIVATION NETWORKS IN QUESTION: PAIN, MEMORY, TACTILE STIMULATION, INTEROCEPTION, ATTENTION, MOTOR EXECUTION, EMOTIONS AND REWARD. ALE MAPS WERE COMPUTED AT AN FDR-CORRECTED THRESHOLD OF $P < 0.05$; MINIMUM CLUSTER DIMENSION $K>100MM^3$ AND VISUALIZED USING BRAINVOYAGER QX 2.1.

FIG S2 AREAS OF OVERLAP BETWEEN PAIN NETWORK AND MEMORY, TACTILE STIMULATION, INTEROCEPTION, ATTENTION, MOTOR EXECUTION, EMOTIONS AND REWARD NETWORKS.

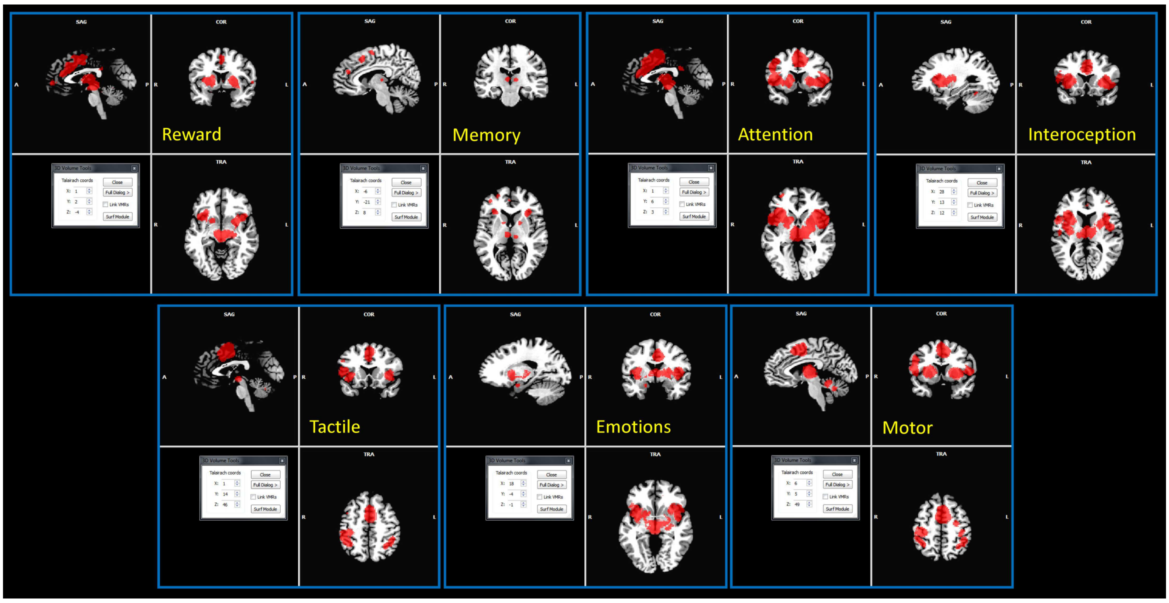

ALE MAPS WERE COMPUTED AT AN FDR-CORRECTED THRESHOLD OF $P < 0.05$; MINIMUM CLUSTER DIMENSION K>100MM3

FIG S3 CONJUNCTION ANALYSIS OF PAIN, MEMORY, TACTILE STIMULATION, INTEROCEPTION, ATTENTION, MOTOR EXECUTION, EMOTIONS AND REWARD NETWORKS.

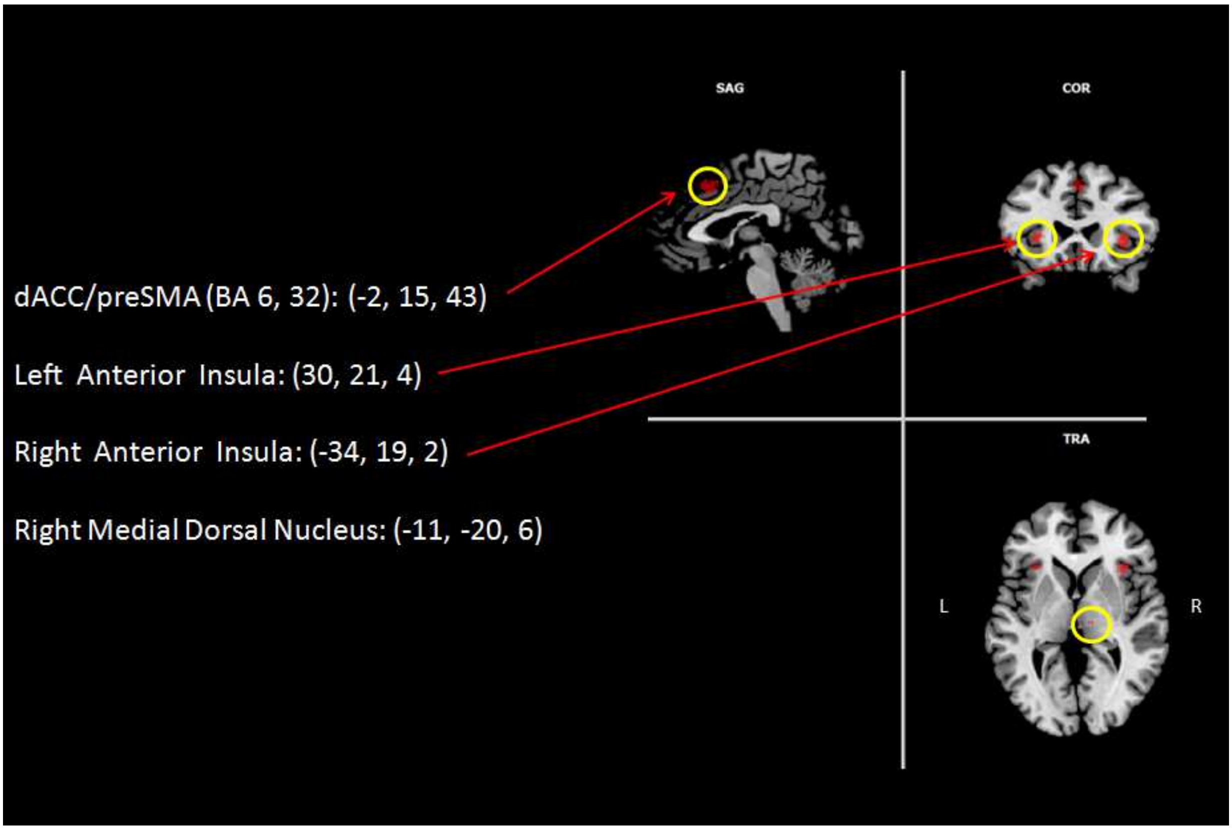

FIG S4 "HUB" NETWORK METAANALYTIC CONNECTIVITY

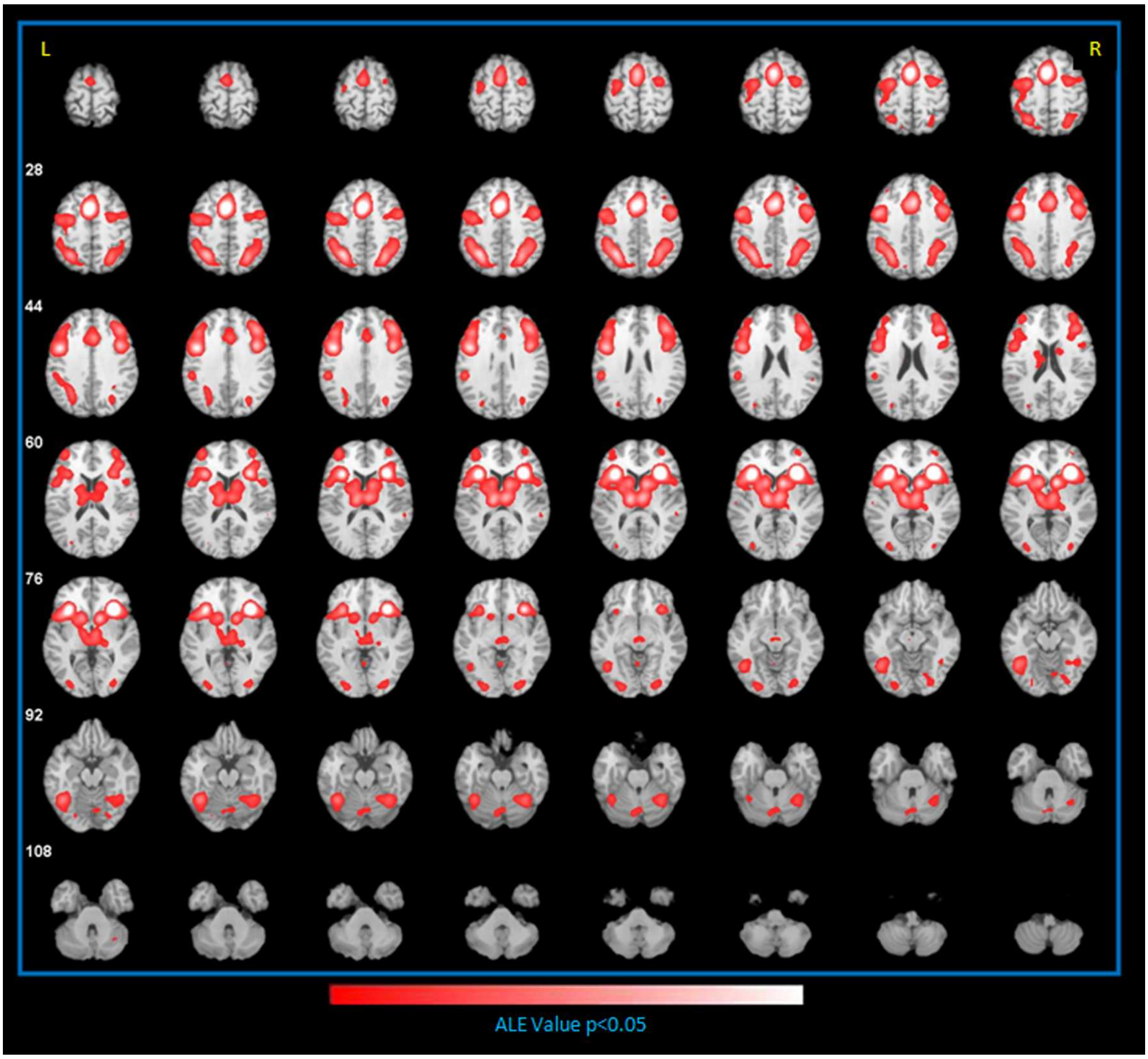

ALE MAPS WERE COMPUTED AT AN FDR-CORRECTED THRESHOLD OF $P < 0.05$; MINIMUM CLUSTER DIMENSION K>100MM3

FIG S5 AREAS OF OVERLAP BETWEEN THE "HUB" NETWORK" META-ANALYTIC CONNECTIVITY AND THE FRONTOPARIETAL ATTENTIONAL NETWORK

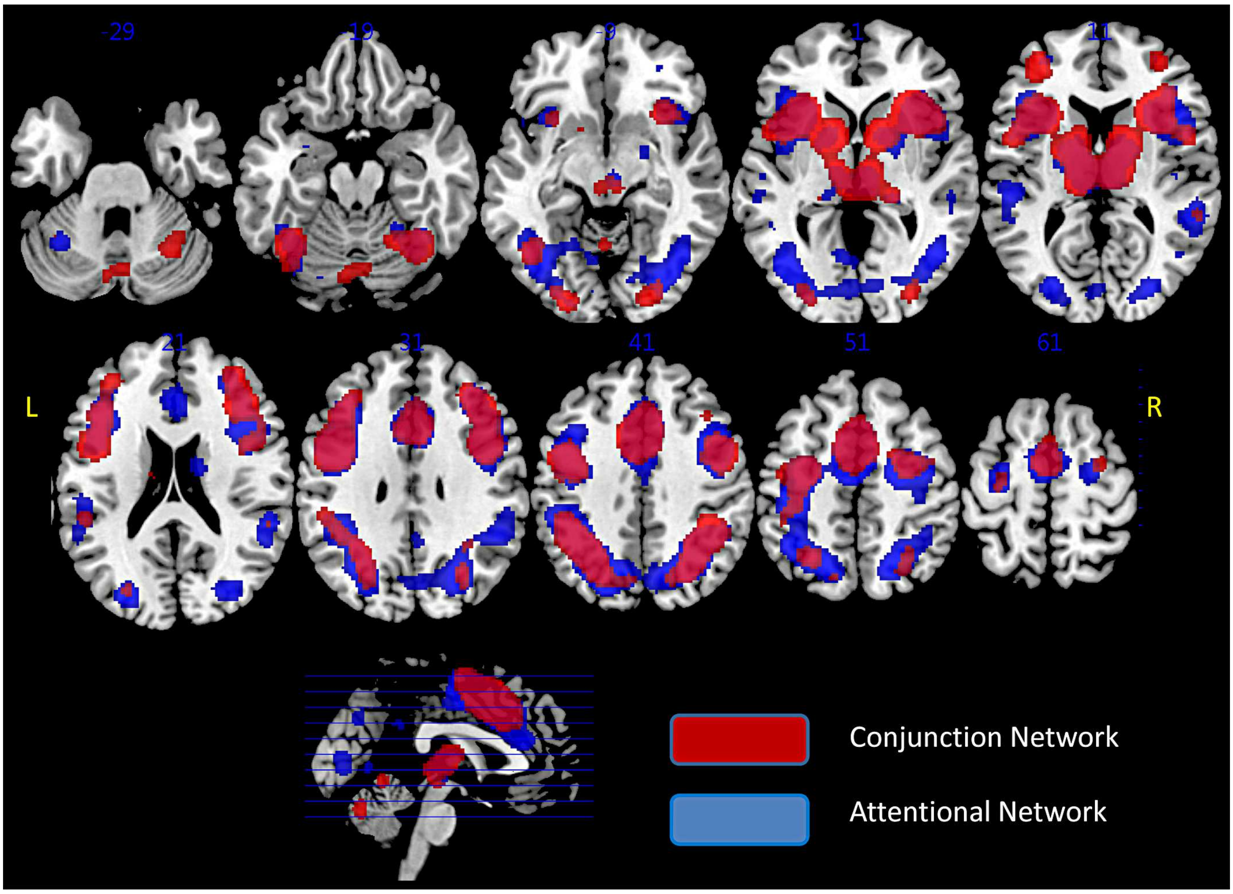

ALE MAPS WERE COMPUTED AT AN FDR-CORRECTED THRESHOLD OF P < 0.05; MINIMUM CLUSTER DIMENSION K>100MM³ 100MM³ AND VISUALIZED USING MRICRON (HTTP://WWW.CABIATL.COM/MRICRO/MRICRON/INDEX.HTM).

FIG S6 NETWORK MODEL OF ANTERIOR INSULA FUNCTION.

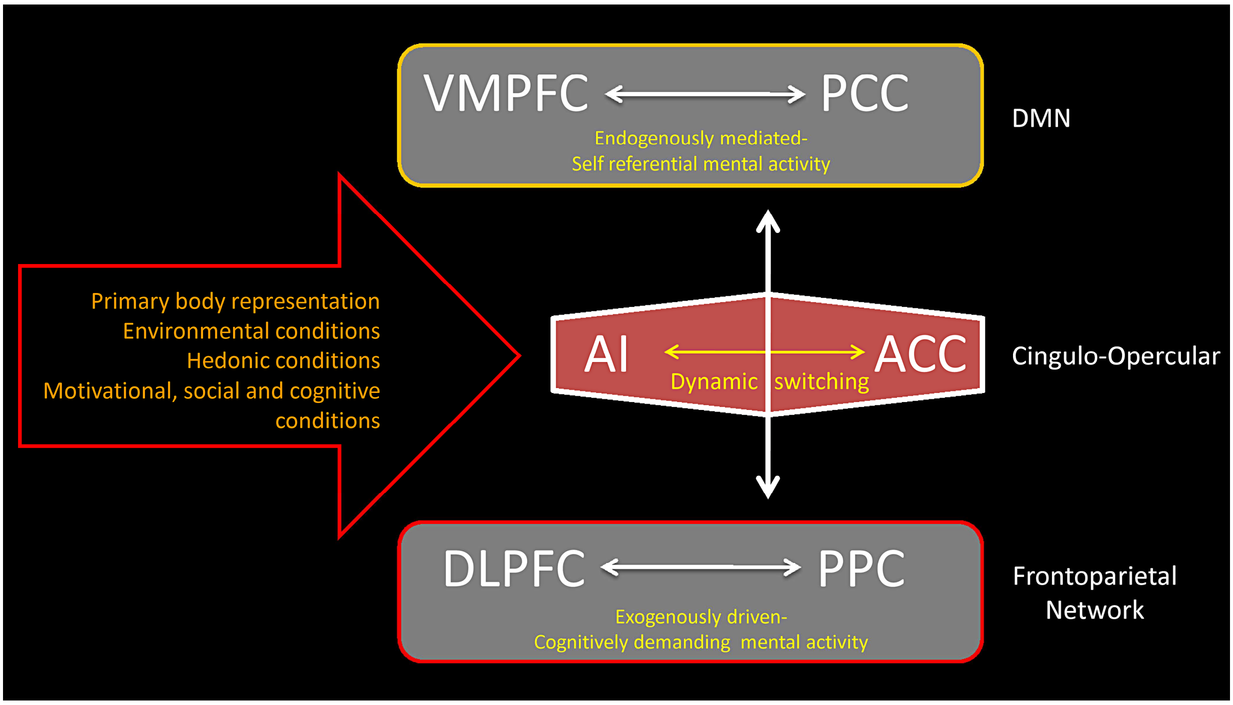

[1]

FIG S7 MODEL OF THE INSULAR STIMULUS INTEGRATION

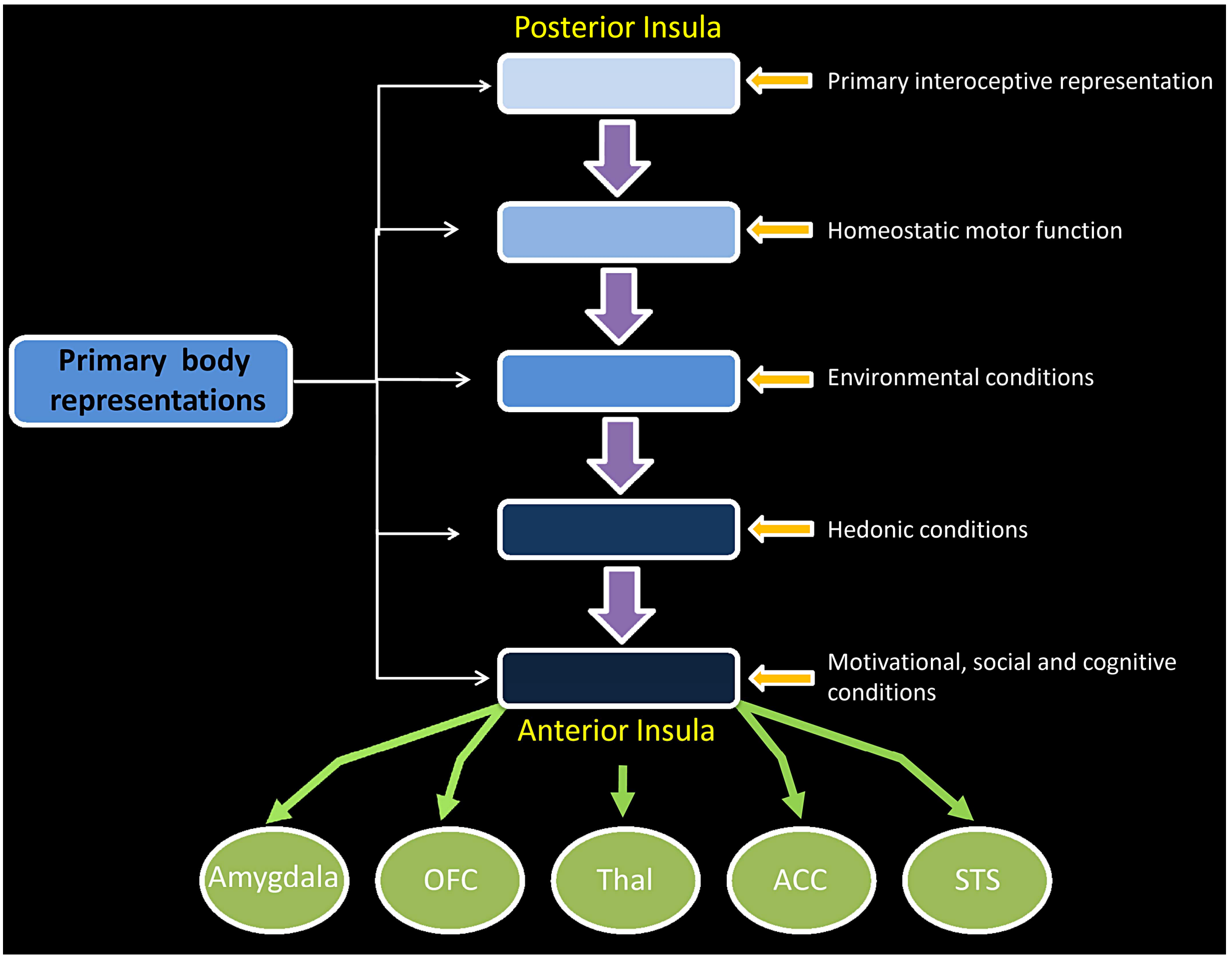

[2,3,4]

FIG S8 AREAS OF OVERLAP BETWEEN PAIN NETWORK AND TACTILE STIMULATION NETWORK.

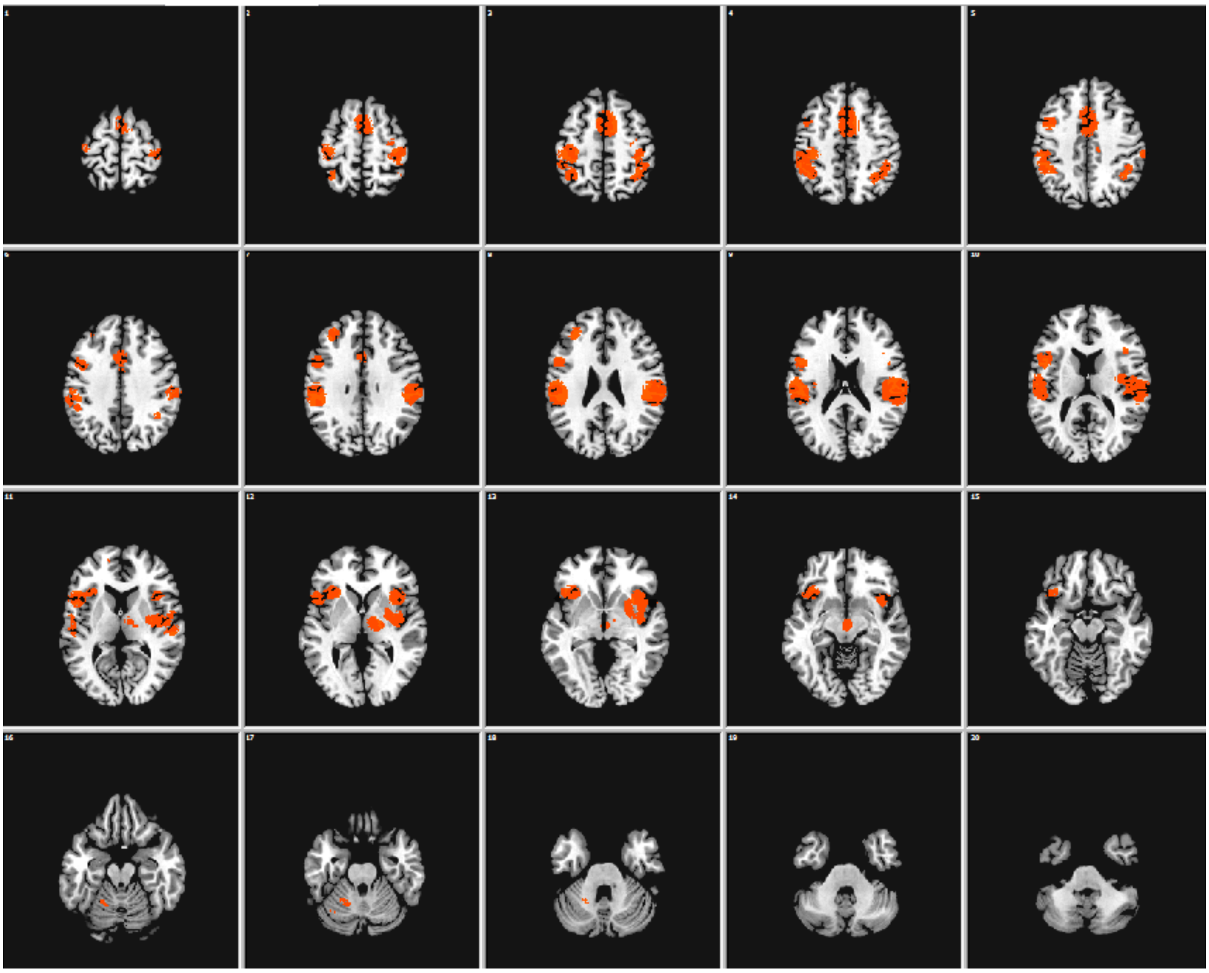

ALE MAPS WERE COMPUTED AT AN FDR-CORRECTED THRESHOLD OF P < 0.05; MINIMUM CLUSTER DIMENSION K>100MM3

FIG S9 AREAS OF OVERLAP BETWEEN PAIN NETWORK AND ATTENTION NETWORK.

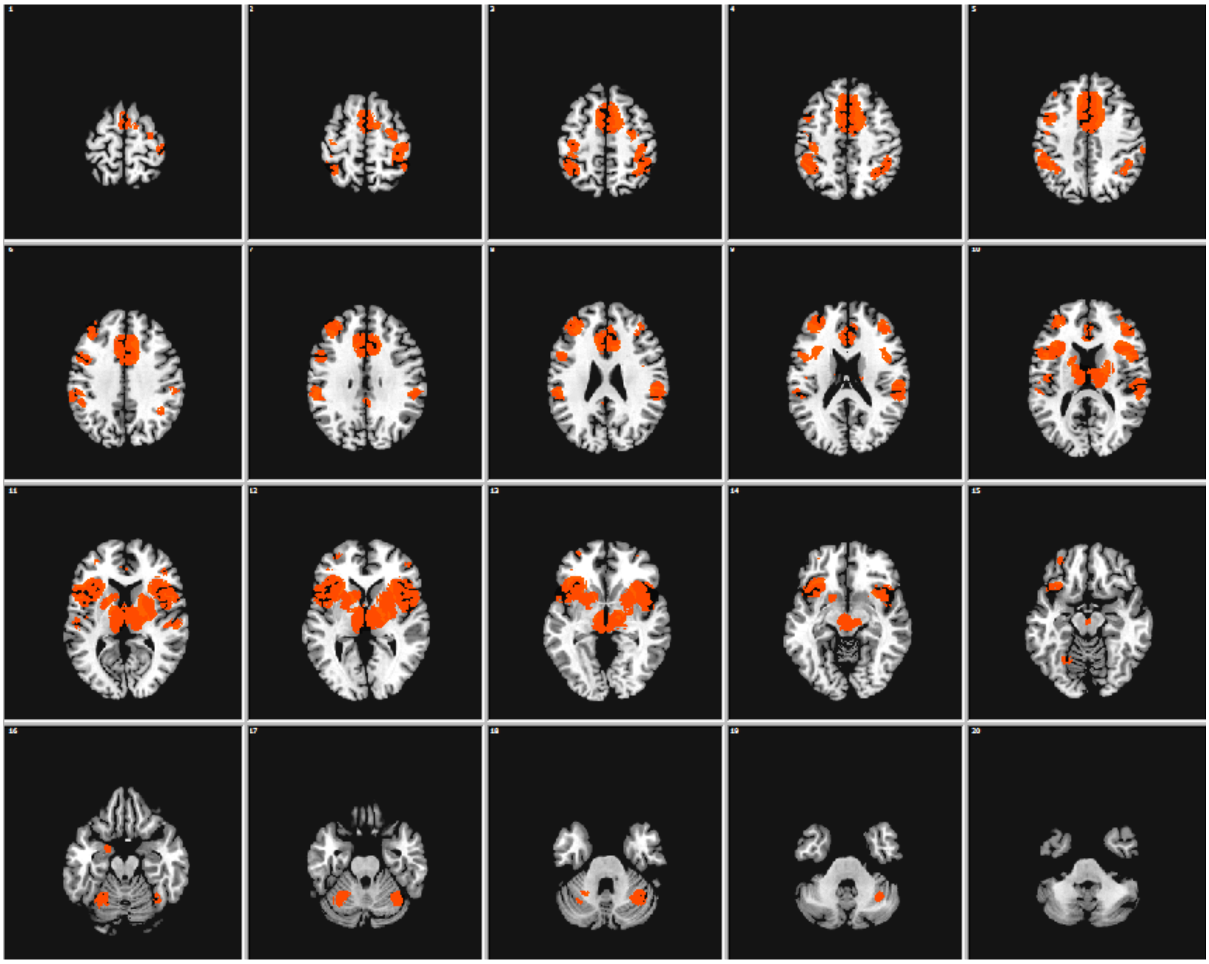

ALE MAPS WERE COMPUTED AT AN FDR-CORRECTED THRESHOLD OF P < 0.05; MINIMUM CLUSTER DIMENSION K>100MM3

FIG S10 AREAS OF OVERLAP BETWEEN PAIN NETWORK AND EMOTION NETWORK.

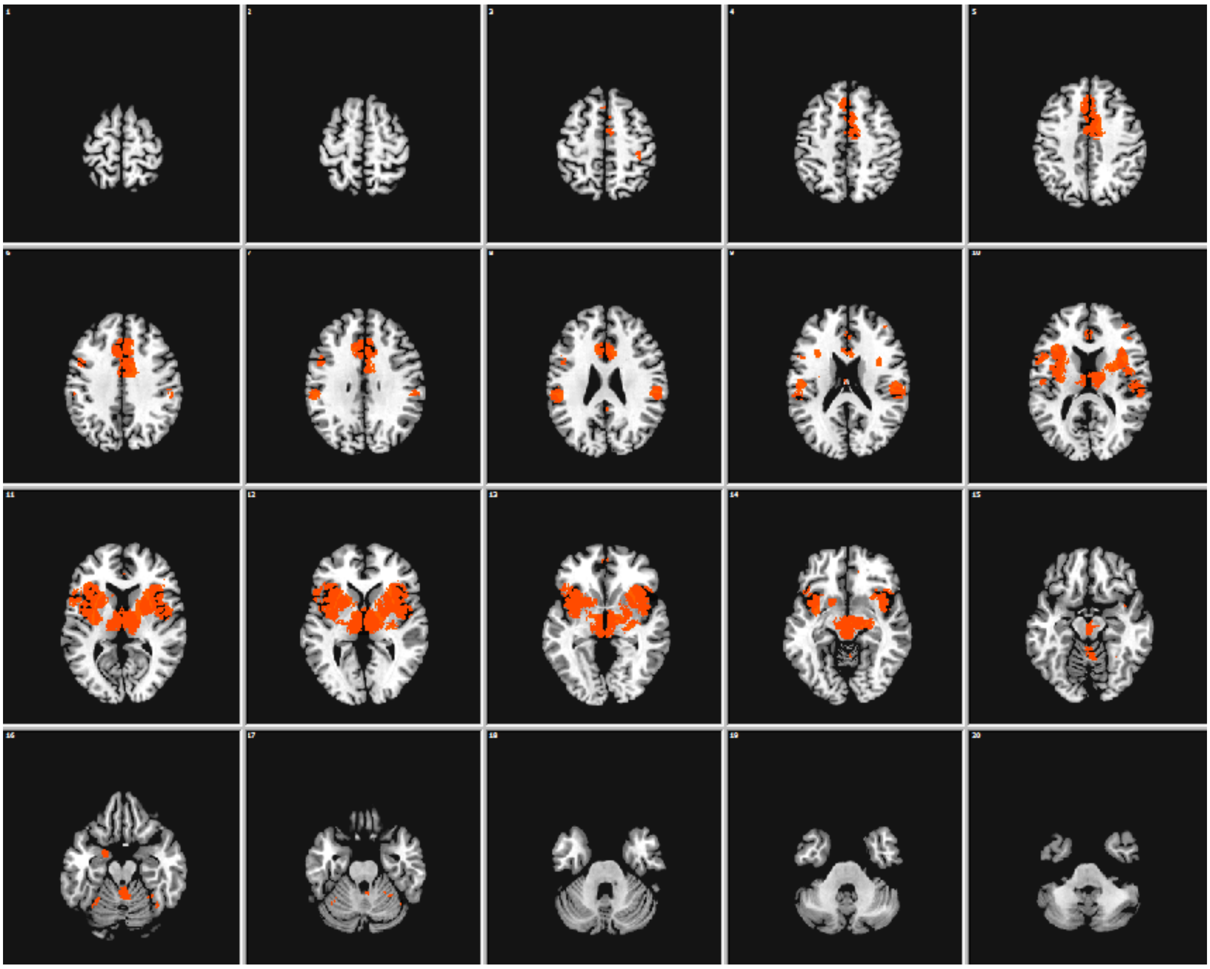

ALE MAPS WERE COMPUTED AT AN FDR-CORRECTED THRESHOLD OF P < 0.05; MINIMUM CLUSTER DIMENSION K>100MM3

FIG S11 AREAS OF OVERLAP BETWEEN PAIN NETWORK AND INTEROCEPTION NETWORK.

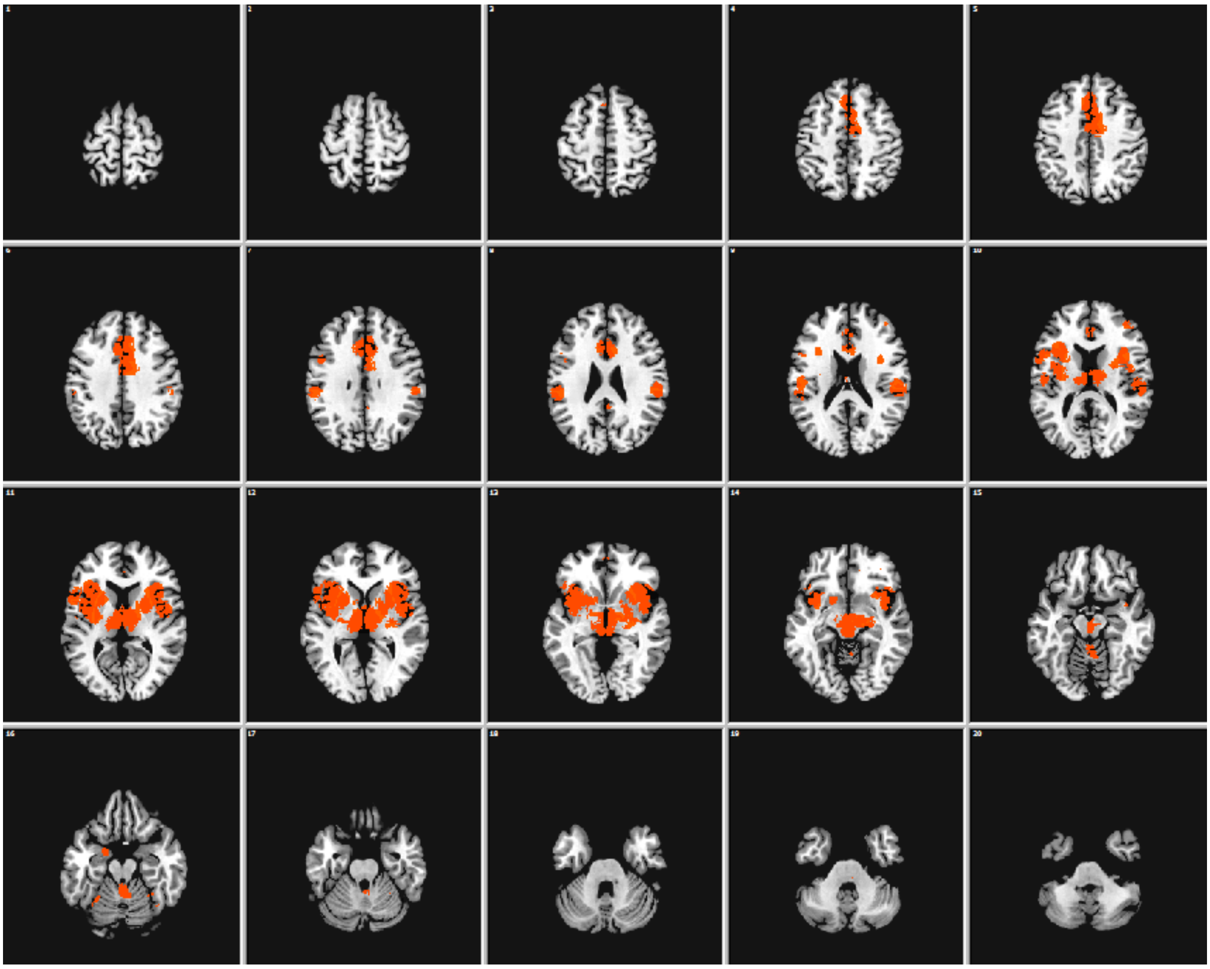

ALE MAPS WERE COMPUTED AT AN FDR-CORRECTED THRESHOLD OF P < 0.05; MINIMUM CLUSTER DIMENSION K>100MM3

FIG S12 AREAS OF OVERLAP BETWEEN PAIN NETWORK AND MEMORY NETWORK.

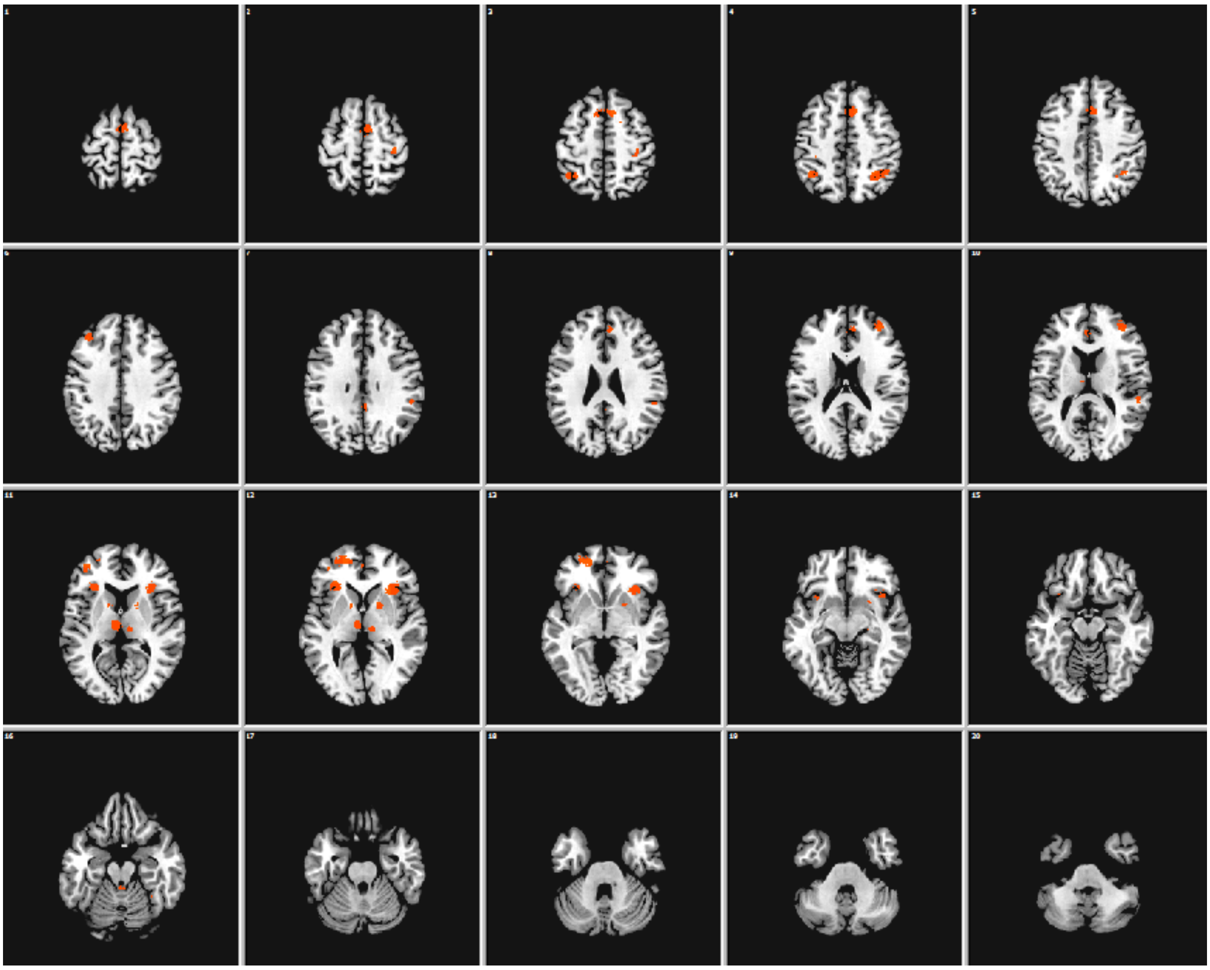

ALE MAPS WERE COMPUTED AT AN FDR-CORRECTED THRESHOLD OF P < 0.05; MINIMUM CLUSTER DIMENSION K>100MM3

FIG S13 AREAS OF OVERLAP BETWEEN PAIN NETWORK AND MOTOR NETWORK.

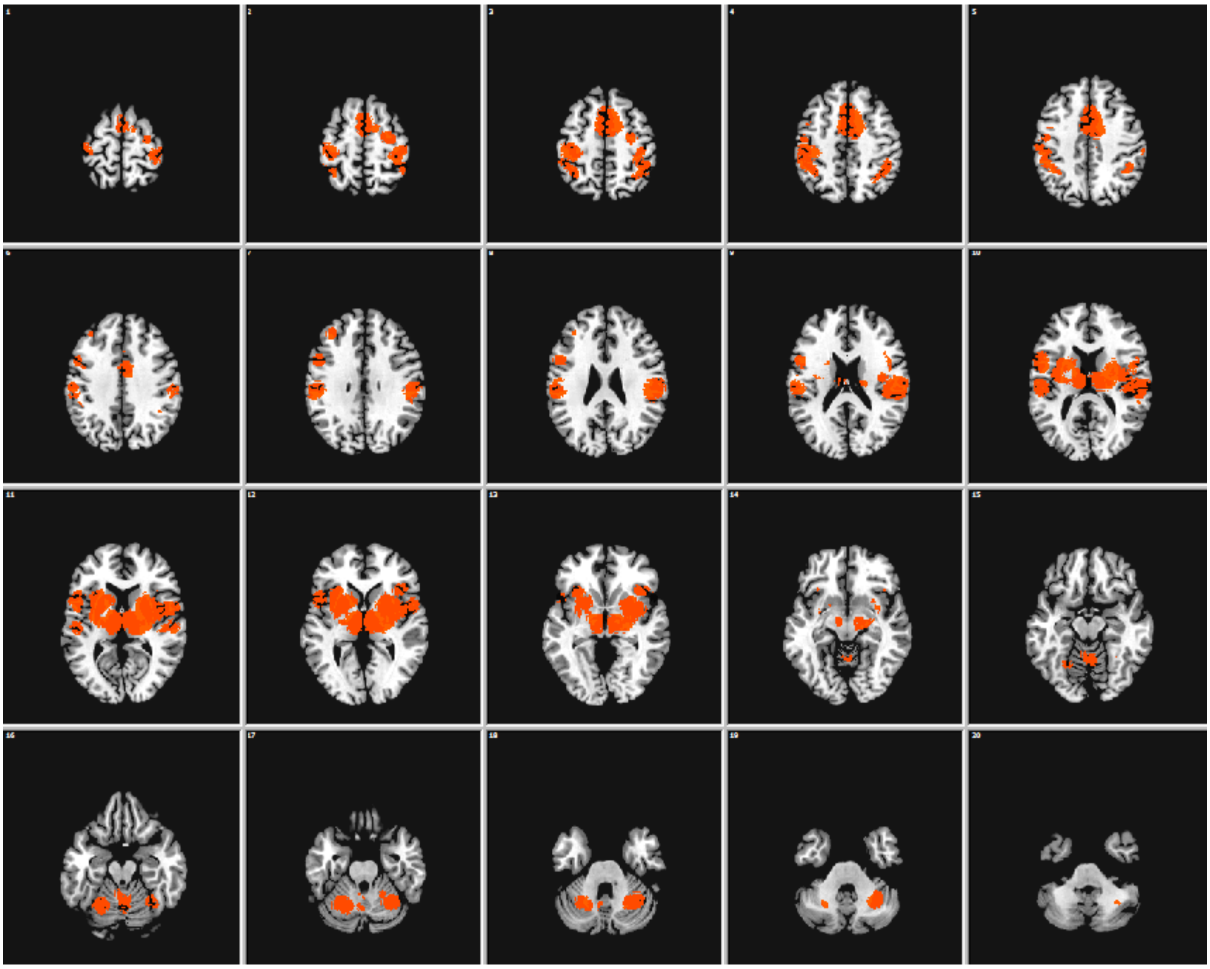

ALE MAPS WERE COMPUTED AT AN FDR-CORRECTED THRESHOLD OF P < 0.05; MINIMUM CLUSTER DIMENSION K>100MM3

<u>REFERENCES</u>

1. Uddin LQ, Menon V (2009) The anterior insula in autism: under-connected and under-examined. Neurosci Biobehav Rev 33: 1198-1203.
2. Craig AD (2009) How do you feel--now? The anterior insula and human awareness. Nat Rev Neurosci 10: 59-70.
3. Mesulam MM, Mufson EJ (1982) Insula of the old world monkey. III: Efferent cortical output and comments on function. J Comp Neurol 212: 38-52.
4. Mufson EJ, Mesulam MM (1982) Insula of the old world monkey. II: Afferent cortical input and comments on the claustrum. J Comp Neurol 212: 23-37.
