# Supplementary material for: Shared “Core” Areas between the Pain and Other Task-Related Networks
Source: PLoS One. 2012 Aug 10;7(8):e41929. doi: 10.1371/journal.pone.0041929 (PMC3416807; doi:10.1371/journal.pone.0041929)
Supplement: Table S1 — Additional tables showing the papers involved in the study and the results of each topic-related metaanalysis. (PDF) [file pone.0041929.s002.pdf]

SUPPLEMENTARY TABLES

TAB. S1 PAPERS INCLUDED IN THE METAANALYSIS INVOLVING ATTENTION

1st Auth.	Journal	Year
**Abler**	Journal of Psychiatric Research	2007
**Altshuler**	Biological Psychiatry	2005
**Anderson**	Journal of Cognitive Neuroscience	2000
**Armony**	Neuroreport	2001
**Aron**	Journal of Neurophysiology	2004
**Aron**	Journal of Neuroscience	2006
**Arrington**	Journal of Cognitive Neuroscience	2000
**Asahi**	European Archives of Psychiatry and Clinical Neuroscience	2004
**Astafiev**	Journal of Neuroscience	2003
**Banich**	Journal of Cognitive Neuroscience	2000
**Banich**	Cognitive Brain Research	2000
**Banich**	Progress in Brain Research	2001
**Bantick**	Brain	2002
**Barch**	Archives of General Psychiatry	2001
**Barch**	Cerebral Cortex	2001
**Bayless**	Neuroscience Letters	2006
**Beauchamp**	NeuroImage	2001
**Beauregard**	Journal of Cognitive Neuroscience	1997
**Bedwell**	International Journal of Neuroscience	2005
**Bellgrove**	Neuropsychologia	2004
**Bench**	Neuropsychologia	1993
**Berman**	Neuropsychologia	1995
**Bermpohl**	Human Brain Mapping	2006
**Binkofski**	Journal of Neurophysiology	2002
**Bird**	NeuroImage	2006

**Blair**	NeuroImage	2007
**Blaxton**	Journal of Neuroscience	1996
**Brass**	NeuroImage	2001
**Brass**	Cerebral Cortex	2002
**Brass**	Journal of Cognitive Neuroscience	2004
**Braver**	Cerebral Cortex	2001
**Braver**	Neuron	2003
**Brown**	Journal of the International Neuropsychological Society	1999
**Buchel**	Journal of Neuroscience	1999
**Buchel**	Neuron	1998
**Burton**	Cerebral Cortex	1999
**Bush**	Human Brain Mapping	1998
**Cabeza**	Neuropsychologia	2003
**Calhoun**	Human Brain Mapping	2006
**Cao**	Brain Research	2008
**Carlsson**	NeuroImage	2006
**Carlsson**	Journal of Cognitive Neuroscience	2000
**Carter**	NeuroImage	1995
**Carter**	Proceedings of the National Academy of Sciences	2000
**Carter**	American Journal of Psychiatry	2001
**Chandrasekhar**	NeuroImage	2008
**Chen**	Biological Psychiatry	2006
**Christoff**	Cortex	2004
**Coderre**	Brain and Language	2008
**Connolly**	Journal of Neurophysiology	2005
**Cools**	Journal of Neuroscience	2004
**Corbetta**	Journal of Neuroscience	1991
**Corbetta**	Nature Neuroscience	2000
**Corbetta**	Neuron	1998
**Corbetta**	Journal of Neuroscience	1993

**Coull**	Science	2004
**Critchley**	Brain	2000
**Critchley**	Neuron	2002
**Critchley**	Nature Neuroscience	2004
**Cross**	Journal of Cognitive Neuroscience	2007
**Dalton**	Journal of Cognitive Neuroscience	2005
**Dassonville**	NeuroImage	2001
**de**	Human Brain Mapping	2001
**de**	Neuropsychologia	2000
**Derbyshire**	Experimental Brain Research	1998
**Derrfuss**	Human Brain Mapping	2005
**Dichter**	NeuroImage	2007
**DiGirolamo**	Neuroreport	2001
**Dove**	Cognitive Brain Research	2000
**Dreher**	Cerebral Cortex	2003
**Dreher**	NeuroImage	2002
**Dreher**	Proceedings of the National Academy of Sciences	2002
**Dunsmoor**	NeuroImage	2008
**Durston**	NeuroImage	2003
**Ellermann**	Journal of Magnetic Resonance	1998
**Elliott**	Archives of General Psychiatry	2002
**Engels**	Psychophysiology	2007
**Ewbank**	NeuroImage	2009
**Eyler**	Psychiatry Research	2004
**Falconer**	Journal of Psychiatry and Neuroscience	2008
**Fan**	NeuroImage	2003
**Fassbender**	Cognitive Brain Research	2004
**Ferrandez**	NeuroImage	2003
**Fiehler**	European Journal of Neuroscience	2004
**Fink**	Brain	1997

**Fischer**	Behavioral Neuroscience	2000
**Fitzgerald**	Biological Psychiatry	2005
**Forstmann**	Journal of Cognitive Neuroscience	2008
**Frankenstein**	NeuroImage	2001
**Fredrikson**	Psychophysiology	1998
**Garavan**	NeuroImage	2002
**Garavan**	Proceedings of the National Academy of Sciences	1999
**Garavan**	NeuroImage	2003
**Garavan**	Cerebral Cortex	2000
**George**	Human Brain Mapping	1994
**Georgiou-Karistianis**	Neuropsychologia	2007
**Giesbrecht**	NeuroImage	2003
**Gitelman**	NeuroImage	2002
**Gitelman**	Brain	1999
**Goldberg**	NeuroImage	1998
**Gomot**	Brain	2008
**Gur**	Human Brain Mapping	2007
**Gurd**	Brain	2002
**Habel**	Neuropsychologia	2007
**Hazeltine**	Neuropsychologia	2003
**Heckers**	American Journal of Psychiatry	2004
**Herwig**	NeuroImage	2007
**Hester**	Journal of Cognitive Neuroscience	2004
**Hoeft**	Journal of Psychiatric Research	2008
**Holland**	American Journal of Psychiatry	2005
**Holmes**	Schizophrenia Research	2005
**Hopfinger**	Nature Neuroscience	2000
**Horn**	Neuropsychologia	2003
**Horovitz**	Magnetic Resonance Imaging	2002
**Iidaka**	Journal of Cognitive Neuroscience	2000

**Indovina**	Experimental Brain Research	2001
**Isenberg**	Proceedings of the National Academy of Sciences	1999
**Jager**	Psychopharmacology	2006
**Jensen**	Neuron	2003
**Jeong**	Psychiatry Research	2005
**Jimura**	NeuroImage	2004
**Johansen-Berg**	Neuroreport	2000
**Johnson-Frey**	Cerebral Cortex	2005
**Jonides**	Journal of Cognitive Neuroscience	1997
**Jordan**	NeuroImage	2001
**Kaladjian**	Bipolar Disorders	2009
**Kaladjian**	Psychiatry Research	2009
**Kastner**	Journal of Neurophysiology	1999
**Kawashima**	Brain Research	1996
**Kelly**	European Journal of Neuroscience	2004
**Kerns**	American Journal of Psychiatry	2005
**Kerns**	NeuroImage	2006
**Kiehl**	Psychophysiology	2000
**Kiehl**	Schizophrenia Research	2001
**Kim**	NeuroImage	1999
**Kimberg**	Cognitive Brain Research	2000
**Knutson**	Social Neuroscience	2006
**Konishi**	Nature Neuroscience	1998
**Konishi**	Proceedings of the National Academy of Sciences	2002
**Konishi**	European Journal of Neuroscience	1998
**Konishi**	Journal of Neuroscience	2003
**Konishi**	Brain	1999
**Koski**	Experimental Brain Research	1999
**Koski**	Cerebral Cortex	2002
**Kringelbach**	NeuroImage	2003

**Kronhaus**	Bipolar Disorders	2006
**Kumari**	Psychiatry Research	2003
**LaBar**	NeuroImage	1999
**LaBar**	Neuron	1998
**Lagopoulos**	Neuroreport	2007
**Laurens**	Brain	2003
**Laurens**	Schizophrenia Research	2005
**Law**	Acta Physiologica Scandinavica	1997
**Lee**	Cerebral Cortex	2008
**Leung**	Cerebral Cortex	2000
**Li**	Neuroscience	2008
**Liddle**	Human Brain Mapping	2001
**Liu**	NeuroImage	2004
**Luks**	NeuroImage	2002
**MacDonald**	Science	2000
**MacDonald**	American Journal of Psychiatry	2005
**MacDonald**	Journal of Abnormal Psychology	2003
**Maclin**	Neuroreport	2001
**Maguire**	NeuroImage	2003
**Malhi**	Journal of Affective Disorders	2007
**Maltby**	NeuroImage	2005
**Marois**	Neuron	2000
**Marois**	Neuron	2000
**Matsumoto**	Experimental Brain Research	2004
**McIntosh**	Science	1999
**Mead**	Journal of the International Neuropsychological Society	2002
**Mendrek**	British Journal of Psychiatry	2004
**Menon**	Human Brain Mapping	2001
**Milham**	NeuroImage	2003
**Milham**	Brain and Cognition	2002

**Milham**	Cognitive Brain Research	2001
**Milham**	Human Brain Mapping	2005
**Milham**	Cognitive Brain Research	2003
**Mitchell**	Neuropsychologia	2003
**Mitterschiffthaler**	Psychological Medicine	2008
**Monchi**	Journal of Neuroscience	2001
**Monchi**	Journal of Neuroscience	2004
**Morris**	NeuroImage	2004
**Morris**	NeuroImage	2001
**Mostofsky**	Cognitive Brain Research	2003
**Mottaghy**	Psychiatry Research	2007
**Muller**	American Journal of Psychiatry	2003
**Nagahama**	Brain	1996
**Nagahama**	Cerebral Cortex	2001
**Nagahama**	Experimental Brain Research	1997
**Nakahara**	Science	2002
**Nakao**	Psychiatry Research	2005
**Nelson**	Bipolar Disorders	2007
**Nobre**	Brain	1997
**Nobre**	NeuroImage	2002
**Nobre**	Nature Neuroscience	1999
**Norris**	NeuroImage	2002
**O'Leary**	Neuropsychopharmacology	2002
**O'Leary**	Neuroreport	2000
**Omori**	Neuroscience Research	1999
**Ortigue**	Journal of Cognitive Neuroscience	2007
**Pardo**	Proceedings of the National Academy of Sciences	1990
**Parris**	Journal of Cognitive Neuroscience	2007
**Paus**	Journal of Neurophysiology	1993
**Peelen**	NeuroImage	2004

**Perlstein**	Biological Psychiatry	2003
**Peterson**	Cognitive Brain Research	2002
**Peterson**	Biological Psychiatry	1999
**Petit**	Human Brain Mapping	1999
**Peyron**	Brain	1999
**Pfefferbaum**	NeuroImage	2001
**Phelps**	Neuron	2004
**Phelps**	Nature Neuroscience	2001
**Phillips**	NeuroImage	2004
**Pierno**	Neuroscience Letters	2008
**Poldrack**	Journal of Neuroscience	2005
**Pollmann**	Human Brain Mapping	2000
**Potenza**	American Journal of Psychiatry	2003
**Price**	Human Brain Mapping	2005
**Ragland**	Neuropsychology	1998
**Ramnani**	Journal of Neurophysiology	2000
**Rauch**	Biological Psychiatry	2007
**Ravnkilde**	Journal of Clinical and Experimental Neuropsychology	2002
**Righi**	Journal of Cognitive Neuroscience	2009
**Rogers**	Journal of Cognitive Neuroscience	2000
**Rosen**	Journal of Cognitive Neuroscience	1999
**Roth**	Biological Psychiatry	2007
**Roth**	Neuroreport	2006
**Rubia**	NeuroImage	2001
**Rubia**	Schizophrenia Research	2001
**Rubia**	NeuroImage	2003
**Rubia**	Human Brain Mapping	2006
**Ruff**	NeuroImage	2001
**Ruge**	Journal of Magnetic Resonance Imaging	2003
**Rushworth**	Journal of Neuroscience	2001

**Rushworth**	Journal of Neurophysiology	2002
**Salo**	Biological Psychiatry	2009
**Schiltz**	Cortex	2001
**Schirmer**	NeuroImage	2004
**Schneider**	Schizophrenia Research	2007
**Schreurs**	Journal of Neurophysiology	1997
**Seidler**	Science	2002
**Sevostianov**	International Journal of Neuroscience	2002
**Shaywitz**	NeuroImage	2001
**Shulman**	Journal of Neuroscience	1999
**Shulman**	Proceedings of the National Academy of Sciences	2001
**Simmonds**	Neuropsychologia	2007
**Simon**	Neuron	2002
**Simon**	NeuroImage	2004
**Smith**	Human Brain Mapping	2004
**Sommer**	Acta Neurobiologiae Experimentalis	2008
**Steel**	Neuroreport	2001
**Stern**	Brain Research	2007
**Stevens**	Magnetic Resonance Imaging	2000
**Strakowski**	Neuropsychopharmacology	2004
**Strangman**	Neurorehabilitation and Neural Repair	2005
**Sturm**	Neuropsychologia	1999
**Sugiura**	NeuroImage	2001
**Suskauer**	Journal of the American Academy of Child and Adolescent Psychiatry	2009
**Suskauer**	Journal of Cognitive Neuroscience	2008
**Swainson**	Journal of Cognitive Neuroscience	2003
**Sylvester**	Neuropsychologia	2003
**Szameitat**	Journal of Cognitive Neuroscience	2002
**Tamm**	Journal of the American Academy of Child and Adolescent Psychiatry	2002
**Tang**	Journal of Cognitive Neuroscience	2006

**Taylor**	NeuroImage	1997
**Thiel**	NeuroImage	2004
**Ullsperger**	NeuroImage	2001
**Ursu**	Psychological Science	2003
**Valet**	Pain	2004
**van**	NeuroImage	2001
**van**	Hearing Research	2003
**Vandenberghe**	NeuroImage	2001
**Vannini**	NeuroImage	2004
**Veit**	Neuroscience Letters	2002
**Vink**	Human Brain Mapping	2005
**Vouloumanos**	Journal of Cognitive Neuroscience	2001
**Vuilleumier**	Neuron	2001
**Wagner**	Biological Psychiatry	2006
**Watanabe**	NeuroImage	2002
**Weiss**	Psychiatry Research	2003
**Wessa**	American Journal of Psychiatry	2007
**Whalen**	Biological Psychiatry	1998
**Wicker**	Brain Research Reviews	2003
**Wild**	Psychiatry Research	2003
**Winterer**	NeuroImage	2002
**Wittfoth**	NeuroImage	2006
**Wittfoth**	Brain Research	2008
**Wong**	Journal of Speech, Language, and Hearing Research	2008
**Wood**	Journal of Cognitive Neuroscience	2003
**Yucel**	American Journal of Psychiatry	2002
**Yucel**	Archives of General Psychiatry	2007
**Zysset**	NeuroImage	2001

TAB S2 PAPERS INCLUDED IN THE METAANALYSIS INVOLVING PAIN

1st Auth.	Journal	Year
**Adler**	Anesthesia and Analgesia	1997
**Bantick**	Brain	2002
**Becerra**	Magnetic Resonance in Medicine	1999
**Becerra**	Neuron	2001
**Bingel**	Pain	2002
**Bingel**	NeuroImage	2003
**Bornhovd**	Brain	2002
**Botvinick**	NeuroImage	2005
**Carlsson**	NeuroImage	2006
**Casey**	Journal of Neurophysiology	1996
**Casey**	Journal of Neurophysiology	2001
**Chandrasekhar**	NeuroImage	2008
**Cheng**	Current Biology	2007
**Coan**	Psychological Science	2006
**Coghill**	Journal of Neuroscience	1994
**Coghill**	Journal of Neurophysiology	2001
**Coghill**	Journal of Neurophysiology	1999
**Coghill**	Proceedings of the National Academy of Sciences	2003
**de**	Oral Surgery, Oral Medicine, Oral Pathology, Oral Radiology, and Endodontics	2006
**Derbyshire**	Experimental Brain Research	1998
**Derbyshire**	NeuroImage	2002
**Derbyshire**	Pain	1997
**Derbyshire**	Journal of Pain	2002
**Derbyshire**	Pain	1998
**Farrell**	Proceedings of the National Academy of Sciences	2006
**Frankenstein**	NeuroImage	2001
**Gelnar**	NeuroImage	1999

**Geuze**	Archives of General Psychiatry	2007
**Giesecke**	Arthritis & Rheumatism	2004
**Gu**	NeuroImage	2007
**Hui**	NeuroImage	2005
**Iadarola**	Brain	1998
**Ibinson**	Anesthesiology	2004
**Jackson**	Neuropsychologia	2006
**Jackson**	NeuroImage	2005
**Jones**	Annals of the Rheumatic Diseases	1997
**Kulkami**	European Journal of Neuroscience	2005
**Lamm**	Journal of Cognitive Neuroscience	2007
**Lorenz**	Neuron	2002
**Maihofner**	European Journal of Neuroscience	2007
**Mochizuki**	NeuroImage	2007
**Moriguchi**	Cerebral Cortex	2007
**Nemoto**	Neuroreport	2003
**Paulson**	Pain	1998
**Petrovic**	Pain	2000
**Petrovic**	NeuroImage	2002
**Petrovic**	Science	2002
**Peyron**	Brain	1999
**Ploner**	Journal of Neurophysiology	2000
**Remy**	NeuroImage	2003
**Salomons**	Journal of Neuroscience	2004
**Schmahl**	Archives of General Psychiatry	2006
**Seifert**	Journal of Neuroscience	2009
**Singer**	Science	2004
**Singer**	Science	2004
**Smith**	British Journal of Psychiatry	2002
**Stoeter**	NeuroImage	2007

**Strigo**	Journal of Neurophysiology	2003
**Svensson**	European Journal of Pain	1998
**Svensson**	Journal of Neurophysiology	1997
**Tolle**	Annals of Neurology	1999
**Tracey**	Neuroscience Letters	2000
**Valet**	Pain	2004
**Veit**	Neuroscience Letters	2002
**Xu**	Neuroreport	1997

TAB S3 PAPERS INCLUDED IN THE METAANALYSIS INVOLVING EMOTION

1st Auth.	Journal	Year
**Abe**	Journal of Cognitive Neuroscience	2007
**Abe**	Cerebral Cortex	2008
**Abel**	Neuroreport	2003
**Abler**	NeuroImage	2006
**Abler**	Journal of Psychiatric Research	2007
**Abler**	Psychopharmacology	2007
**Adcock**	Neuron	2006
**Akitsuki**	NeuroImage	2003
**Aleman**	PLoS ONE	2008
**Altshuler**	Bipolar Disorders	2008
**Amir**	Biological Psychiatry	2005
**Anand**	Biological Psychiatry	2005
**Armony**	Neuroreport	2001
**Aron**	Journal of Neurophysiology	2005
**Aron**	Journal of Neurophysiology	2004
**Ashwin**	Neuropsychologia	2007
**Azari**	European Journal of Neuroscience	2001
**Baker**	Psychological Medicine	1997
**Ballard**	NeuroImage	2009

**Bartels**	NeuroImage	2004
**Bartels**	Neuroreport	2000
**Bartolo**	Journal of Cognitive Neuroscience	2006
**Baumgartner**	Brain Research	2006
**Beauregard**	Journal of Cognitive Neuroscience	1997
**Beauregard**	Neuroreport	1998
**Beauregard**	Neuroreport	2006
**Beblo**	Psychological Medicine	2006
**Beck**	Biological Psychiatry	2009
**Beneventi**	Scandinavian Journal of Psychology	2007
**Bermpohl**	Human Brain Mapping	2006
**Bickel**	Journal of Neuroscience	2009
**Bjork**	Journal of Neuroscience	2004
**Bjork**	Behavioural Brain Research	2007
**Blair**	Brain	1999
**Blair**	Journal of Neuroscience	2006
**Blair**	NeuroImage	2007
**Blood**	Proceedings of the National Academy of Sciences	2001
**Bocher**	NeuroImage	2001
**Bolla**	NeuroImage	2005
**Bolla**	Cerebral Cortex	2004
**Breiter**	Neuron	1996
**Breiter**	Neuron	2001
**Bremner**	Journal of Affective Disorders	2007
**Britton**	NeuroImage	2006
**Britton**	NeuroImage	2006
**Britton**	Biological Psychiatry	2005
**Brown**	Neuroreport	2004
**Brunia**	Experimental Brain Research	2000
**Buchanan**	Cognitive Brain Research	2000

**Buchel**	Neuron	1998
**Budhani**	NeuroImage	2007
**Butler**	Neuroscience	2007
**Bystritsky**	Neuroreport	2001
**Calder**	European Journal of Neuroscience	2008
**Camara**	Frontiers in Human Neuroscience	2009
**Canli**	Neuroreport	1998
**Canli**	Proceedings of the National Academy of Sciences	2002
**Canli**	Neuroreport	2004
**Canli**	Proceedings of the National Academy of Sciences	2005
**Carlsson**	NeuroImage	2006
**Carr**	Proceedings of the National Academy of Sciences	2003
**Caseras**	Biological Psychiatry	2007
**Cato**	Journal of Cognitive Neuroscience	2004
**Chandrasekhar**	NeuroImage	2008
**Chang**	Archives of General Psychiatry	2004
**Chen**	Biological Psychiatry	2006
**Clark**	Neuron	2009
**Coan**	Psychological Science	2006
**Cooper**	NeuroImage	2008
**Cox**	Journal of Neuroscience	2005
**Crespo-Facorro**	Journal of American Medical Association	2001
**Critchley**	Brain	2000
**Critchley**	Human Brain Mapping	2000
**Critchley**	Neuron	2002
**Crosson**	Neuroreport	1999
**Cunningham**	Journal of Cognitive Neuroscience	2004
**Dalton**	Nature Neuroscience	2005
**Dalton**	Journal of Cognitive Neuroscience	2005
**Damasio**	Nature Neuroscience	2000

**Dapretto**	Nature Neuroscience	2006
**Davidson**	American Journal of Psychiatry	2003
**Deckersbach**	Bipolar Disorders	2008
**Deeley**	Biological Psychiatry	2007
**Deeley**	British Journal of Psychiatry	2006
**Dickstein**	Bipolar Disorders	2007
**Dilger**	Neuroscience Letters	2003
**Dillon**	Psychophysiology	2007
**Dohnel**	Neuropsychologia	2008
**Dolan**	NeuroImage	2000
**Dolan**	NeuroImage	1996
**Dolan**	Proceedings of the National Academy of Sciences	2001
**Dolcos**	Journal of Neuroscience	2006
**Dolcos**	NeuroImage	2004
**Dougherty**	Biological Psychiatry	1999
**Drobyshevsky**	NeuroImage	2006
**Eippert**	Human Brain Mapping	2007
**Elliott**	Journal of Neuroscience	2000
**Elliott**	Archives of General Psychiatry	2002
**Elliott**	NeuroImage	2004
**Elliott**	Journal of Neuroscience	2003
**Elliott**	Neuropsychologia	1997
**Elliott**	Psychological Medicine	1998
**Elliott**	Biological Psychiatry	2004
**Engelmann**	PLoS ONE	2009
**Engels**	Psychophysiology	2007
**Epstein**	American Journal of Psychiatry	2006
**Ernst**	Neuropsychologia	2004
**Ernst**	NeuroImage	2005
**Ethofer**	NeuroImage	2006

**Ethofer**	Neuroreport	2006
**Ewbank**	NeuroImage	2009
**Fales**	Biological Psychiatry	2008
**Fales**	Journal of Affective Disorders	2009
**Fenker**	European Journal of Neuroscience	2005
**Finger**	NeuroImage	2006
**Fischer**	Behavioral Neuroscience	2000
**Fitzgerald**	Neuroscience Letters	2004
**Flaisch**	NeuroImage	2009
**Flores-Gutierrez**	International Journal of Psychophysiology	2007
**Foland**	Psychiatry Research	2008
**Fox**	Human Brain Mapping	2002
**Frangou**	European Psychiatry	2008
**Fredrikson**	Psychophysiology	1998
**Frey**	European Journal of Neuroscience	2000
**Fu**	Archives of General Psychiatry	2004
**Fu**	American Journal of Psychiatry	2007
**Fu**	Biological Psychiatry	2008
**Fu**	Biological Psychiatry	2008
**Fukui**	NeuroImage	2005
**Galvan**	Developmental Science	2007
**Gamer**	Human Brain Mapping	2007
**Gandour**	Human Brain Mapping	2003
**Ganis**	Cerebral Cortex	2003
**Garrett**	NeuroImage	2006
**Gemar**	Depression	1996
**George**	Human Brain Mapping	1994
**George**	American Journal of Psychiatry	1995
**George**	Biological Psychiatry	1996
**George**	Journal of Neuropsychiatry and Clinical Neuroscience	1993

**George**	Archives of Neurology	1996
**Goldin**	NeuroImage	2005
**Goldin**	Biological Psychiatry	2008
**Goldstein**	American Journal of Psychiatry	2007
**Goossens**	Psychiatry Research	2007
**Gorno-Tempini**	NeuroImage	2001
**Gotlib**	Neuroreport	2005
**Grandjean**	Nature Neuroscience	2005
**Grimm**	NeuroImage	2006
**Grimm**	Biological Psychiatry	2008
**Grosbras**	Cerebral Cortex	2006
**Guroglu**	NeuroImage	2008
**Gusnard**	Proceedings of the National Academy of Sciences	2003
**Gusnard**	Proceedings of the National Academy of Sciences	2001
**Habel**	NeuroImage	2005
**Habel**	Neuropsychologia	2007
**Hall**	Neuroreport	2004
**Hall**	American Journal of Psychiatry	2003
**Hamann**	Nature Neuroscience	1999
**Harenski**	NeuroImage	2006
**Hariri**	Neuroreport	2000
**Hariri**	Biological Psychiatry	2003
**Haruno**	Journal of Neuroscience	2004
**Hassel**	Bipolar Disorders	2008
**Hennenlotter**	NeuroImage	2005
**Herpertz**	Journal of Child Psychology and Psychiatry	2008
**Herwig**	Psychiatry Research	2007
**Herwig**	NeuroImage	2007
**Hoffman**	Psychopharmacology	2008
**Holt**	Schizophrenia Research	2006

**Hou**	Brain Research	2007
**Huettel**	Neuron	2006
**Huss**	NeuroImage	2008
**Hutcherson**	Social Cognitive and Affective Neuroscience	2008
**Iaria**	Human Brain Mapping	2008
**Imaizumi**	Neuroreport	1997
**Isenberg**	Proceedings of the National Academy of Sciences	1999
**Iwase**	NeuroImage	2002
**Jabbi**	NeuroImage	2007
**Jacobsen**	NeuroImage	2006
**Janata**	Cerebral Cortex	2009
**Jensen**	Neuron	2003
**Jogia**	British Journal of Psychiatry	2008
**Johnstone**	Journal of Neuroscience	2007
**Juckel**	NeuroImage	2006
**Junghofer**	Neuroreport	2006
**Kawabata**	Journal of Neurophysiology	2004
**Keedwell**	Biological Psychiatry	2005
**Keightley**	Social Cognitive and Affective Neuroscience	2007
**Kensinger**	Neuropsychologia	2007
**Kesler-West**	Cognitive Brain Research	2001
**Kim**	PLoS Biology	2006
**Kimbrell**	Biological Psychiatry	1999
**Kirsch**	NeuroImage	2003
**Kirsch**	Neuroscience Letters	2006
**Knutson**	Journal of Neuroscience	2005
**Knutson**	Journal of Neuroscience	2001
**Knutson**	Social Neuroscience	2006
**Knutson**	NeuroImage	2000
**Knutson**	NeuroImage	2003

**Knutson**	Neuroreport	2001
**Knutson**	Neuron	2004
**Knutson**	Biological Psychiatry	2008
**Koch**	Neuropsychologia	2007
**Koeneke**	Behavioral and Brain Functions	2008
**Kosslyn**	Neuroreport	1996
**Kotz**	Brain and Language	2003
**Kozel**	Behavioral Neuroscience	2004
**Kozel**	Biological Psychiatry	2005
**Kozel**	Journal of Neuropsychiatry and Clinical Neuroscience	2004
**Kramer**	NeuroImage	2007
**Kuchinke**	NeuroImage	2005
**Kulkami**	European Journal of Neuroscience	2005
**LaBar**	Neuron	1998
**Ladurner**	Social Neuroscience	2006
**Lagopoulos**	Neuroreport	2007
**Lamm**	Journal of Cognitive Neuroscience	2007
**Lane**	American Journal of Psychiatry	1997
**Lane**	Neuropsychologia	1997
**Lane**	Neuropsychologia	1999
**Lane**	Neuroreport	1997
**Langleben**	Human Brain Mapping	2005
**Langleben**	NeuroImage	2002
**Lanius**	Biological Psychiatry	2002
**Lanius**	Biological Psychiatry	2003
**Lanius**	Biological Psychiatry	2005
**Lawrence**	Biological Psychiatry	2004
**Lawrence**	Biological Psychiatry	2007
**Lawrence**	NeuroImage	2006
**Lee**	Cognitive Behavioral Neurology	2004

**Lee**	Cerebral Cortex	2008
**Lee**	Social Cognitive and Affective Neuroscience	2006
**Lee**	Brain and Cognition	2009
**Leibenluft**	Biological Psychiatry	2004
**Lennox**	Psychological Medicine	2004
**Liberzon**	Neuropsychopharmacology	2000
**Liotti**	Biological Psychiatry	2000
**Lissek**	PLoS ONE	2008
**Little**	Brain and Cognition	2006
**Liu**	Journal of Neuroscience	2007
**LoPresti**	Journal of Neuroscience	2008
**Lorberbaum**	Neuroreport	2004
**Maddock**	Human Brain Mapping	2003
**Maddock**	Psychiatry Research	1997
**Malhi**	Bipolar Disorders	2007
**Malhi**	Journal of Affective Disorders	2007
**Malhi**	European Journal of Neuroscience	2004
**Malhi**	Bipolar Disorders	2004
**Malhi**	Bipolar Disorders	2005
**Marjoram**	NeuroImage	2006
**Markowitsch**	Cortex	2003
**Marsh**	NeuroImage	2007
**Martin-Soelch**	European Journal of Neuroscience	2001
**Martin-Soelch**	European Journal of Neuroscience	2003
**Martin-Solch**	Experimental Brain Research	2001
**Mataix-Cols**	Archives of General Psychiatry	2004
**Mathews**	Journal of Cognitive Neuroscience	2004
**Mathiak**	Human Brain Mapping	2006
**Mayberg**	American Journal of Psychiatry	1999
**McClure**	Neuron	2004

**McClure**	Science	2004
**Mitchell**	European Journal of Neuroscience	2006
**Mitchell**	Neuropsychologia	2003
**Mitchell**	British Journal of Psychiatry	2004
**Mitterschiffthaler**	Neuroreport	2003
**Mitterschiffthaler**	Psychological Medicine	2008
**Mobbs**	Science	2007
**Mock**	Journal of Clinical Psychiatry	1997
**Mohamed**	Radiology	2006
**Monterosso**	Human Brain Mapping	2007
**Morris**	Brain	1998
**Morris**	Neuropsychologia	1999
**Morris**	Nature	1996
**Morris**	NeuroImage	2004
**Morris**	NeuroImage	2001
**Najib**	American Journal of Psychiatry	2004
**Nakamura**	Journal of Neurophysiology	1999
**Nakamura**	Neuroreport	1998
**Nieuwenhuis**	European Journal of Neuroscience	2005
**Nieuwenhuis**	NeuroImage	2005
**Nitschke**	NeuroImage	2004
**Noriuchi**	Biological Psychiatry	2008
**Nunez**	NeuroImage	2005
**O'Doherty**	Neuropsychologia	2003
**Ochsner**	Journal of Cognitive Neuroscience	2002
**Ochsner**	Journal of Cognitive Neuroscience	2004
**Ogino**	Cerebral Cortex	2007
**Ortigue**	Journal of Cognitive Neuroscience	2007
**Ortigue**	NeuroImage	2007

**Paradiso**	American Journal of Psychiatry	1999
**Paradiso**	American Journal of Psychiatry	1997
**Pardo**	American Journal of Psychiatry	1993
**Partiot**	Neuroreport	1995
**Pavuluri**	Biological Psychiatry	2007
**Payer**	Drug and Alcohol Dependence	2008
**Pelchat**	NeuroImage	2004
**Pelletier**	Neuroreport	2003
**Pessoa**	Proceedings of the National Academy of Sciences	2002
**Phan**	Biological Psychiatry	2005
**Phan**	Academic Radiology	2005
**Phelps**	Nature Neuroscience	2001
**Phillips**	Nature	1997
**Phillips**	Proceedings of the Royal Society of London. Series B. Biological Sciences	1998
**Phillips**	Psychiatry Research	1998
**Phillips**	NeuroImage	2004
**Piefke**	Brain	2003
**Piefke**	Human Brain Mapping	2005
**Pierce**	Brain	2004
**Pietrini**	American Journal of Psychiatry	2000
**Pochon**	Proceedings of the National Academy of Sciences	2002
**Pourtois**	Cortex	2005
**Preuschoff**	Neuron	2006
**Rama**	NeuroImage	2001
**Ramasubbu**	Canadian Journal of Psychiatry	2007
**Ramnani**	Cerebral Cortex	2003
**Ramnani**	NeuroImage	2004
**Ranote**	Neuroreport	2004
**Reiman**	American Journal of Psychiatry	1997
**Reiss**	PLoS ONE	2008

**Remijnse**	Archives of General Psychiatry	2006
**Rilling**	Biological Psychiatry	2007
**Rilling**	Neuroreport	2004
**Rolls**	European Journal of Neuroscience	2007
**Royet**	Journal of Neuroscience	2000
**Sabatinelli**	Journal of Neurophysiology	2007
**Sachdev**	Neuropsychologia	2008
**Sailer**	NeuroImage	2007
**Samanez-Larkin**	Nature Neuroscience	2007
**Sanjuan**	Psychiatry Research	2007
**Scheres**	Biological Psychiatry	2007
**Schienle**	International Journal of Psychophysiology	2005
**Schienle**	Neuroscience Letters	2005
**Schiller**	Journal of Neuroscience	2008
**Schirmer**	NeuroImage	2004
**Schnell**	Journal of Psychiatric Research	2007
**Schwartz**	Brain	2008
**Sergerie**	Schizophrenia Bulletin	2009
**Shamosh**	Psychological Science	2008
**Shapira**	Biological Psychiatry	2003
**Sharot**	Nature Neuroscience	2004
**Shin**	Biological Psychiatry	2000
**Shin**	American Journal of Psychiatry	1999
**Simpson**	Journal of Cognitive Neuroscience	2000
**Singer**	Science	2004
**Singer**	Science	2004
**Small**	Brain	2001
**Sommer**	Acta Neurobiologiae Experimentalis	2008
**Spence**	Neuroreport	2001
**Spence**	NeuroImage	2008

**Spreckelmeyer**	Social Cognitive and Affective Neuroscience	2009
**Sprengelmeyer**	Proceedings of the Royal Society of London. Series B. Biological Sciences	1998
**Stark**	NeuroImage	2007
**Stark**	Biological Psychology	2005
**Steele**	NeuroImage	2004
**Stoeter**	NeuroImage	2007
**Strathearn**	Pediatrics	2008
**Straube**	NeuroImage	2007
**Surguladze**	Biological Psychiatry	2005
**Takahashi**	NeuroImage	2006
**Tapert**	Archives of General Psychiatry	2003
**Taylor**	NeuroImage	1998
**Taylor**	Neuropsychologia	2000
**Taylor**	NeuroImage	2003
**Teasdale**	American Journal of Psychiatry	1999
**Thut**	Neuroreport	1997
**Tobler**	Journal of Neurophysiology	2007
**Tremblay**	Archives of General Psychiatry	2005
**Ueda**	Neuroreport	2003
**Ullsperger**	Journal of Neuroscience	2003
**Vanderwal**	NeuroImage	2008
**Vollm**	NeuroImage	2006
**Volz**	NeuroImage	2003
**Volz**	NeuroImage	2004
**Vuilleumier**	Neuron	2001
**Wang**	Journal of the American Academy of Child and Adolescent Psychiatry	2004
**Wang**	Psychiatry Research	2008
**Whalen**	Biological Psychiatry	1998
**Whalen**	Journal of Neuroscience	1998
**Wicker**	Neuron	2003

**Wild**	Psychiatry Research	2003
**Wildgruber**	NeuroImage	2005
**Wildgruber**	NeuroImage	2002
**Williams**	NeuroImage	2001
**Winston**	NeuroImage	2003
**Winston**	Neuropsychologia	2007
**Wittmann**	Experimental Brain Research	2007
**Wrase**	NeuroImage	2007
**Wrase**	NeuroImage	2007
**Wrase**	Neuroscience Letters	2003
**Wright**	Neuroreport	2002
**Xu**	Brain Research	2009
**Yacubian**	Journal of Neuroscience	2006
**Yang**	Journal of the American Academy of Child and Adolescent Psychiatry	2003
**Zald**	Proceedings of the National Academy of Sciences	1997
**Zald**	Proceedings of the National Academy of Sciences	2002
**Zatorre**	Neuroreport	2000
**Zink**	Neuron	2004

TAB S4 PAPERS INCLUDED IN THE METAANALYSIS INVOLVING INTEROCEPTION

1st Auth.	Journal	Year
**Abler**	Psychopharmacology	2007
**Arnow**	Brain	2002
**Athwal**	Brain	2001
**Aziz**	Journal of Neuroscience	2000
**Baicy**	Proceedings of the National Academy of Sciences	2007
**Beauregard**	Journal of Neuroscience	2001
**Blok**	Brain	1997
**Blok**	Brain	1998
**Blok**	Journal of Comparative Neurology	1997

**Bocher**	NeuroImage	2001
**Brannan**	Proceedings of the National Academy of Sciences	2001
**Britton**	NeuroImage	2006
**Colebatch**	Journal of Physiology	1991
**Cornier**	American Journal of Clinical Nutrition	2007
**Critchley**	Nature Neuroscience	2004
**de**	Journal of Neurophysiology	2003
**Del**	American Journal of Clinical Nutrition	2002
**Denton**	Proceedings of the National Academy of Sciences	1999
**Denton**	Proceedings of the National Academy of Sciences	1999
**Desseilles**	NeuroImage	2006
**Egan**	Proceedings of the National Academy of Sciences	2003
**Evans**	Journal of Physiology	1999
**Farrell**	Proceedings of the National Academy of Sciences	2006
**Ferretti**	NeuroImage	2005
**Fink**	Journal of Applied Physiology	1996
**Fukuyama**	Neuroreport	1996
**Gizewski**	Experimental Brain Research	2006
**Griffiths**	Journal of Urology	2005
**Hobday**	Brain	2001
**Holsen**	NeuroImage	2005
**Huh**	Journal of Sexual Medicine	2008
**Isaev**	Journal of Physiology	2002
**Karama**	Human Brain Mapping	2002
**Killgore**	NeuroImage	2003
**Kim**	International Journal of Impotence Research	2006
**Kuhtz-Buschbeck**	Journal of Urology	2005
**Liotti**	Proceedings of the National Academy of Sciences	2001
**Lotze**	NeuroImage	2001
**Lowell**	NeuroImage	2008

**Matsuura**	Journal of Urology	2002
**McKay**	Journal of Applied Physiology	2003
**McKay**	NeuroImage	2008
**Mehnert**	NeuroImage	2008
**Miyagawa**	NeuroImage	2007
**Moulier**	NeuroImage	2006
**Nour**	Brain	2000
**Nunneley**	Journal of Applied Physiology	2002
**Ortigue**	NeuroImage	2007
**Parsons**	Proceedings of the National Academy of Sciences	2001
**Pelchat**	NeuroImage	2004
**Ponseti**	NeuroImage	2006
**Porubska**	NeuroImage	2006
**Ramsay**	Journal of Physiology	1993
**Redoute**	Human Brain Mapping	2000
**Rosenbaum**	Journal of Clinical Investigations	2008
**Rothemund**	NeuroImage	2007
**Sabatinelli**	Journal of Neurophysiology	2007
**Safron**	Behavioral Neuroscience	2007
**Santel**	Brain Research	2006
**Schaefer**	Biological Psychiatry	2006
**Seseke**	NeuroImage	2006
**Seseke**	NeuroImage	2008
**Shin**	Psychiatry Research	1999
**Simmons**	Cerebral Cortex	2005
**Smeets**	American Journal of Clinical Nutrition	2006
**Stark**	Biological Psychology	2005
**Stoleru**	Archives of Sexual Behavior	1999
**Takahashi**	NeuroImage	2006
**Tataranni**	Proceedings of the National Academy of Sciences	1999

**Tsujimura**	Journal of Urology	2006
**Uher**	Behavioural Brain Research	2006
**Wu**	American Journal of Psychiatry	1999
**Yang**	Korean Journal of Radiology	2004
**Yin**	Journal of Nuclear Medicine	2006
**Zhang**	NeuroImage	2005

TAB S5 PAPERS INCLUDED IN THE METAANALYSIS INVOLVING MEMORY

1st Auth.	Journal	Year
**Achim**	Journal of Cognitive Neuroscience	2005
**Achim**	Archives of General Psychiatry	2007
**Addis**	Neuropsychologia	2007
**Addis**	NeuroImage	2006
**Adler**	Bipolar Disorders	2004
**Akine**	Alcoholism: Clinical and Experimental Research	2007
**Allen**	Psychopharmacology	2006
**Altamura**	Psychiatry Research	2007
**Anderson**	Journal of Cognitive Neuroscience	2000
**Anderson**	Brain	1994
**Andreasen**	Human Brain Mapping	2003
**Andreasen**	Proceedings of the National Academy of Sciences	1996
**Andreasen**	American Journal of Psychiatry	1995
**Aron**	Journal of Neurophysiology	2005
**Aron**	Journal of Neurophysiology	2004
**Assaf**	Biological Psychiatry	2006
**Assaf**	Psychiatry Research	2006
**Audoin**	Human Brain Mapping	2005
**Awh**	Psychological Science	1996
**Barch**	Journal of Abnormal Psychology	2002
**Barch**	Archives of General Psychiatry	2001

**Baumann**	Neuroreport	2007
**Beauregard**	Neuroreport	1998
**Beblo**	Psychological Medicine	2006
**Becker**	Human Brain Mapping	1994
**Bedwell**	International Journal of Neuroscience	2005
**Beneventi**	Scandinavian Journal of Psychology	2007
**Bonda**	Proceedings of the National Academy of Sciences	1995
**Bondi**	Neurology	2005
**Bonner-Jackson**	Biological Psychiatry	2005
**Bonner-Jackson**	Biological Psychiatry	2007
**Braver**	NeuroImage	2001
**Braver**	NeuroImage	1997
**Breitenstein**	NeuroImage	2005
**Bremner**	Journal of Affective Disorders	2007
**Britton**	Biological Psychiatry	2005
**Broome**	British Journal of Psychiatry	2009
**Buckner**	NeuroImage	1998
**Buckner**	Journal of Neuroscience	1996
**Bunge**	Brain	2001
**Burianova**	Journal of Cognitive Neuroscience	2007
**Cabeza**	NeuroImage	2002
**Cabeza**	Neuron	1997
**Cabeza**	Proceedings of the National Academy of Sciences	2001
**Cabeza**	Journal of Cognitive Neuroscience	2003
**Cabeza**	Neuropsychologia	2003
**Cabeza**	NeuroImage	2002
**Cabeza**	Neuroreport	1997
**Cairo**	Cognitive Brain Research	2004
**Caldwell**	Behavioral Neuroscience	2005
**Callicott**	Cerebral Cortex	1999

**Callicott**	American Journal of Psychiatry	2003
**Callicott**	Cerebral Cortex	2000
**Camchong**	Biological Psychiatry	2006
**Campanella**	NeuroImage	2001
**Canli**	Proceedings of the National Academy of Sciences	2002
**Cannon**	Archives of General Psychiatry	2005
**Cansino**	Cerebral Cortex	2002
**Carlson**	Cerebral Cortex	1998
**Casey**	NeuroImage	1998
**Chan**	Neuropsychologia	2008
**Chang**	Archives of General Psychiatry	2004
**Chen**	NeuroImage	2004
**Chochon**	Journal of Cognitive Neuroscience	1999
**Chua**	Hippocampus	2007
**Clark**	Human Brain Mapping	2000
**Cohen**	Human Brain Mapping	1994
**Cohen**	Nature	1997
**Crespo-Facorro**	American Journal of Psychiatry	1999
**Crespo-Facorro**	Human Brain Mapping	2001
**Cross**	Journal of Cognitive Neuroscience	2007
**D'Esposito**	NeuroImage	1998
**Dade**	NeuroImage	2001
**Dannhauser**	Cortex	2008
**Daselaar**	NeuroImage	2001
**Daselaar**	NeuroImage	2004
**Daselaar**	Cerebral Cortex	2006
**Daselaar**	Journal of Neurophysiology	2006
**Daumann**	Neuroreport	2003
**de**	Neuropsychologia	2007
**de**	Cognitive Brain Research	2005

**Deckersbach**	Bipolar Disorders	2008
**Denkova**	Neuropsychologia	2006
**Desmond**	NeuroImage	2003
**Dickstein**	Bipolar Disorders	2007
**Dobbins**	Neuropsychologia	2003
**Dohnel**	Neuropsychologia	2008
**Dolan**	NeuroImage	2000
**Dolcos**	Journal of Neuroscience	2006
**Dolcos**	NeuroImage	2004
**Drapier**	Biological Psychiatry	2008
**Drobyshevsky**	NeuroImage	2006
**Druzgal**	Cognitive Brain Research	2001
**Druzgal**	Neuron	2001
**Dupont**	Surgical and Radiologic Anatomy	2002
**Duzel**	Human Brain Mapping	2001
**Duzel**	Proceedings of the National Academy of Sciences	1999
**Eldridge**	Nature Neuroscience	2000
**Eschen**	International Journal of Psychophysiology	2007
**Eyler**	Psychiatry Research	2008
**Fahim**	Brain and Cognition	2004
**Fenker**	European Journal of Neuroscience	2005
**Fiez**	Journal of Neuroscience	1996
**Fischer**	Emotion	2007
**Fitzgerald**	Human Brain Mapping	2008
**Fletcher**	Brain	1996
**Fletcher**	Archives of General Psychiatry	1998
**Fliessbach**	NeuroImage	2006
**Frangou**	European Psychiatry	2008
**Fujii**	NeuroImage	2002
**Ganguli**	Biological Psychiatry	1997

**Garavan**	Microscopy Research and Technique	2000
**Garavan**	Cerebral Cortex	2000
**Garraux**	Journal of Neuroscience	2005
**Goekoop**	NeuroImage	2005
**Goekoop**	Neuropsychopharmacology	2006
**Gould**	NeuroImage	2003
**Grady**	NeuroImage	2001
**Grady**	Proceedings of the National Academy of Sciences	1998
**Grasby**	Brain	1994
**Grosbras**	Cerebral Cortex	2001
**Gruber**	NeuroImage	2003
**Gruber**	Human Brain Mapping	2009
**Gundersen**	Open Neuroimaging Journal	2008
**Halsband**	Behavioural Brain Research	1998
**Halsband**	Behavioural Brain Research	2002
**Halsband**	Journal of Physiology - Paris	2006
**Hamilton**	Human Brain Mapping	2009
**Harvey**	NeuroImage	2005
**Hassabis**	Journal of Neuroscience	2007
**Hasson**	Neuron	2002
**Hautzel**	Neuroscience Letters	2002
**Haxby**	Neuron	1999
**Heckers**	Nature Neuroscience	1998
**Heide**	European Journal of Neuroscience	2001
**Heinze**	Schizophrenia Research	2006
**Henke**	Hippocampus	1997
**Henke**	Proceedings of the National Academy of Sciences	1999
**Henson**	NeuroImage	2002
**Henson**	Journal of Neuroscience	1999
**Herath**	Human Brain Mapping	2001

**Herrmann**	Human Brain Mapping	2001
**Hester**	Neuropsychopharmacology	2009
**Hofer**	American Journal of Psychiatry	2003
**Hofer**	American Journal of Psychiatry	2003
**Hofer**	Brain and Cognition	2007
**Honey**	NeuroImage	2000
**Honey**	Proceedings of the National Academy of Sciences	1999
**Honey**	Schizophrenia Research	2002
**Honey**	Psychological Medicine	2003
**Hooker**	Neuropsychologia	2008
**Hou**	Brain Research	2007
**Hunkin**	Neuropsychologia	2002
**Iidaka**	Journal of Cognitive Neuroscience	2000
**Ino**	Brain Research Bulletin	2004
**Ishai**	Journal of Cognitive Neuroscience	2000
**Ishai**	Proceedings of the National Academy of Sciences	1999
**Jackson**	NeuroImage	2004
**Jacobsen**	Biological Psychiatry	2004
**Jacobsen**	Psychopharmacology	2007
**Jager**	Psychopharmacology	2006
**Jager**	European Neuropsychopharmacology	2007
**Janata**	Cerebral Cortex	2009
**Jansma**	Schizophrenia Research	2004
**Jeong**	Psychiatry Research	2005
**Jernigan**	NeuroImage	1998
**Jessen**	Human Brain Mapping	2002
**Johnson**	Biological Psychiatry	2006
**Johnson**	Cerebral Cortex	2007
**Johnson**	Brain	2002
**Johnson**	Neurobiology of Aging	2006

**Jonides**	Journal of Cognitive Neuroscience	1997
**Kanayama**	Psychopharmacology	2004
**Kapur**	Journal of Cognitive Neuroscience	1996
**Kawashima**	Experimental Brain Research	1998
**Kelley**	Neuron	1998
**Kensinger**	Neuropsychologia	2007
**Kensinger**	Journal of Neuroscience	2006
**Kikyo**	NeuroImage	2004
**Kim**	NeuroImage	2002
**Kim**	American Journal of Psychiatry	2003
**Kindermann**	Schizophrenia Research	2004
**Kirschen**	NeuroImage	2005
**Koch**	Neuroscience	2007
**Koch**	Neuropsychologia	2007
**Kohler**	Acta Psychologica	2000
**Kohler**	Neuropsychologia	1998
**Koppelstaetter**	NeuroImage	2008
**Koshino**	Cerebral Cortex	2008
**Krause**	Brain	1999
**Kubicki**	NeuroImage	2003
**Kumari**	Schizophrenia Research	2006
**LaBar**	NeuroImage	1999
**Lagopoulos**	Journal of Psychiatry and Neuroscience	2007
**Landau**	NeuroImage	2004
**Lange**	NeuroImage	2005
**Langenecker**	Biological Psychiatry	2007
**Lanius**	Biological Psychiatry	2002
**Lanius**	American Journal of Psychiatry	2004
**Lanius**	Psychiatry Research	2007
**Lanius**	Biological Psychiatry	2003

**Lanius**	Biological Psychiatry	2005
**Leaver**	Journal of Neuroscience	2009
**Lee**	Human Brain Mapping	2002
**Lee**	Annals of Neurology	2000
**Lee**	Brain and Cognition	2009
**Lepage**	NeuroImage	2001
**Lepage**	Biological Psychiatry	2006
**Leung**	Journal of Cognitive Neuroscience	2002
**Leveroni**	Journal of Neuroscience	2000
**Levine**	Journal of Cognitive Neuroscience	2004
**Liberzon**	Neuropsychopharmacology	2000
**Lim**	Neuropsychobiology	2008
**Linden**	NeuroImage	2003
**Liu**	Human Brain Mapping	2007
**LoPresti**	Journal of Neuroscience	2008
**Macrae**	Cerebral Cortex	2004
**Mainero**	NeuroImage	2004
**Malhi**	Journal of Affective Disorders	2007
**Malisza**	Pediatric Research	2005
**Mandzia**	Neurobiology of Aging	2009
**Manoach**	Schizophrenia Research	2005
**Manoach**	Biological Psychiatry	2000
**Maquet**	NeuroImage	1996
**Maril**	NeuroImage	2003
**Martinkauppi**	Cerebral Cortex	2000
**Matsuo**	Molecular Psychiatry	2007
**Mayer**	NeuroImage	2007
**Mazard**	Journal of Cognitive Neuroscience	2002
**McNab**	Neuropsychologia	2008
**Meisenzahl**	European Archives of Psychiatry and Clinical Neuroscience	2006

**Mellet**	NeuroImage	2000
**Mellet**	Cerebral Cortex	2002
**Meltzer**	NeuroImage	2005
**Mencl**	Microscopy Research and Technique	2000
**Mendrek**	British Journal of Psychiatry	2004
**Mendrek**	Psychological Medicine	2005
**Menon**	NeuroImage	2001
**Mensebach**	Psychiatry Research	2009
**Meyer-Lindenberg**	American Journal of Psychiatry	2001
**Miller**	Journal of Cognitive Neuroscience	2002
**Monks**	Bipolar Disorders	2004
**Montaldi**	Hippocampus	2006
**Mottaghy**	Experimental Brain Research	1999
**Mu**	Sleep	2005
**Mu**	Sleep	2005
**Murray**	Journal of Neuroscience	2007
**Nakamura**	Neuropsychologia	2001
**Nakao**	Journal of Psychiatric Research	2009
**Neuner**	Brain Research	2007
**Nitschke**	Human Brain Mapping	2004
**Nunez**	NeuroImage	2005
**Nyberg**	Journal of Cognitive Neuroscience	2000
**Nyberg**	Journal of Neuroscience	1996
**Nyberg**	Proceedings of the National Academy of Sciences	1996
**Nystrom**	NeuroImage	2000
**O'Sullivan**	Neuroreport	1995
**Ongur**	Archives of General Psychiatry	2006
**Ongur**	Psychiatry Research	2005
**Otten**	Cerebral Cortex	2007
**Owen**	Proceedings of the National Academy of Sciences	1998

**Owen**	European Journal of Neuroscience	1999
**Padula**	Psychology of Addictive Behaviors	2007
**Paller**	Learning & Memory	2003
**Pardo**	American Journal of Psychiatry	1993
**Pariente**	Annals of Neurology	2005
**Paus**	Journal of Neurophysiology	1993
**Peelen**	Neuron	2006
**Perlstein**	American Journal of Psychiatry	2001
**Perlstein**	Biological Psychiatry	2003
**Pessoa**	Neuron	2002
**Peters**	European Journal of Neuroscience	2007
**Petit**	Journal of Neuroscience	1998
**Petit**	Journal of Neuroscience	1996
**Petrella**	Radiology	2006
**Petrella**	Radiology	2007
**Petrides**	Proceedings of the National Academy of Sciences	1993
**Pfefferbaum**	NeuroImage	2001
**Phillips**	Psychiatry Research	1998
**Piblajamaki**	American Journal of Geriatric Psychiatry	2008
**Piefke**	Brain	2003
**Piefke**	Human Brain Mapping	2005
**Pihlajamaki**	Hippocampus	2003
**Platek**	Human Brain Mapping	2006
**Pochon**	Cerebral Cortex	2001
**Pochon**	Proceedings of the National Academy of Sciences	2002
**Postle**	Cerebral Cortex	2007
**Prado**	Journal of Cognitive Neuroscience	2007
**Prince**	Journal of Neuroscience	2005
**Qin**	NeuroImage	2007
**Quintana**	Biological Psychiatry	2003

**Ragland**	Neuropsychology	2002
**Ragland**	Neuropsychology	1998
**Ragland**	American Journal of Psychiatry	2001
**Ragland**	Schizophrenia Research	2006
**Ragland**	American Journal of Psychiatry	2004
**Ragland**	American Journal of Psychiatry	2005
**Rama**	NeuroImage	2001
**Rand-Giovannetti**	Neurobiology of Aging	2006
**Ranganath**	Neuropsychologia	2003
**Rauch**	Biological Psychiatry	2007
**Reber**	Cognitive Brain Research	2002
**Reiman**	American Journal of Psychiatry	1997
**Ricciardi**	Neuroscience	2006
**Ries**	NeuroImage	2006
**Robinson**	Bipolar Disorders	2009
**Rowe**	Science	2000
**Rypma**	NeuroImage	1999
**Rypma**	Psychology and Aging	2001
**Sabri**	Journal of Nuclear Medicine	2003
**Sailer**	NeuroImage	2007
**Salgado-Pineda**	NeuroImage	2004
**Sanchez-Carrion**	Journal of Neurotrauma	2008
**Savage**	Brain	2001
**Savic**	Neuron	2000
**Schlosser**	Neuroreport	2003
**Schmidt**	Neuropsychologia	2002
**Schmidt**	Human Brain Mapping	2009
**Schneider**	Schizophrenia Research	2007
**Schumacher**	NeuroImage	1996
**Sergerie**	Schizophrenia Bulletin	2009

**Sevostianov**	Human Brain Mapping	2002
**Shamosh**	Psychological Science	2008
**Sharot**	Nature Neuroscience	2004
**Shaw**	NeuroImage	2002
**Shen**	Human Brain Mapping	1999
**Sheridan**	Journal of the American Academy of Child and Adolescent Psychiatry	2007
**Shikata**	Journal of Neurophysiology	2001
**Shikata**	European Journal of Neuroscience	2003
**Shimomura**	Turkish Neruosurgery	2008
**Shin**	Biological Psychiatry	2000
**Shin**	American Journal of Psychiatry	1999
**Simmons**	Cerebral Cortex	2005
**Skosnik**	NeuroImage	2002
**Smith**	Cerebral Cortex	1996
**Smith**	Journal of Clinical Endocrinology and Metabolism	2006
**Sowell**	Neuroreport	2007
**Sperling**	Human Brain Mapping	2001
**Sperling**	NeuroImage	2003
**Sperling**	Journal of Neurology, Neurosurgery, and Psychiatry	2003
**Squire**	Proceedings of the National Academy of Sciences	1992
**Staresina**	Journal of Neuroscience	2006
**Stern**	NeuroImage	2000
**Stevens**	Archives of General Psychiatry	1998
**Suchan**	Hippocampus	2008
**Sugiura**	NeuroImage	2001
**Summerfield**	NeuroImage	2009
**Suzuki**	NeuroImage	2002
**Sweeney**	Journal of Neurophysiology	1996
**Tan**	American Journal of Psychiatry	2006
**Tan**	American Journal of Psychiatry	2005

**Tanaka**	BMC Neurology	2006
**Taylor**	NeuroImage	1998
**Thermenos**	American Journal of Medical Genetics Part B: Neuropsychiatric Genetics	2009
**Thierry**	Human Brain Mapping	2003
**Tsukiura**	Human Brain Mapping	2002
**Uncapher**	Journal of Neuroscience	2005
**Uncapher**	Neuron	2006
**Vaidya**	Neuropsychologia	2002
**van**	Cerebral Cortex	2003
**van**	Nature Neuroscience	2000
**van**	NeuroImage	2003
**Vandekerckhove**	Behavioural Neurology	2005
**Veltman**	NeuroImage	2003
**Vilberg**	Neuropsychologia	2007
**Vinogradov**	Cerebral Cortex	2008
**Volle**	Cerebral Cortex	2005
**Wagner**	Neuroreport	1998
**Walsh**	Biological Psychiatry	2007
**Walter**	Schizophrenia Research	2003
**Walter**	Journal of Affective Disorders	2007
**Watanabe**	NeuroImage	2008
**Weis**	Neuroreport	2004
**Weiss**	Biological Psychiatry	2006
**Werner**	Journal of Affective Disorders	2009
**Wheeler**	Proceedings of the National Academy of Sciences	2000
**Wheeler**	NeuroImage	2004
**Woodard**	Journal of Cognitive Neuroscience	2007
**Woodruff**	Neuropsychologia	2005
**Wykes**	British Journal of Psychiatry	2002
**Xu**	Brain	2009

**Yonelinas**	Journal of Neuroscience	2005
**Yoo**	International Journal of Neuroscience	2005
**Yoon**	Neuroscience Letters	2009
**Zago**	Neuroscience Letters	2002
**Zurowski**	NeuroImage	2002
**Zysset**	Neuroscience Letters	2001
**Zysset**	NeuroImage	2002

TAB S6 PAPERS INCLUDED IN THE METAANALYSIS INVOLVING MOTOR

1st Auth.	Journal	Year
**Aoki**	Experimental Brain Research	2005
**Aramaki**	Cerebral Cortex	2006
**Bengtsson**	European Journal of Neuroscience	2005
**Bestmann**	NeuroImage	2005
**Binkofski**	Human Brain Mapping	2000
**Binkofski**	Journal of Neurophysiology	2002
**Blakemore**	NeuroImage	1999
**Blinkenberg**	Journal of Cerebral Blood Flow and Metabolism	1996
**Blok**	Journal of Comparative Neurology	1997
**Bodegard**	Neuroreport	2000
**Boecker**	Journal of Neurophysiology	1998
**Bookheimer**	Neurology	2000
**Brass**	NeuroImage	2001
**Broome**	British Journal of Psychiatry	2009
**Brown**	Cerebral Cortex	2008
**Calautti**	Stroke	2001
**Carey**	NeuroImage	2000
**Carr**	Proceedings of the National Academy of Sciences	2003
**Catalan**	Brain	1998
**Catalan**	Brain	1999

**Colebatch**	Journal of Neurophysiology	1991
**Corfield**	Journal of Applied Physiology	1999
**Creem-Regehr**	Cognitive Brain Research	2005
**Cunnington**	NeuroImage	2006
**Dapretto**	Nature Neuroscience	2006
**De**	Experimental Brain Research	2005
**Denslow**	Biological Psychiatry	2005
**Dimitrova**	Brain Research Bulletin	2006
**Ding**	Neuroreport	2003
**Drobyshevsky**	NeuroImage	2006
**Dziewas**	NeuroImage	2003
**Ehrsson**	European Journal of Neuroscience	2000
**Ehrsson**	Journal of Neurophysiology	2003
**Fahim**	Brain and Cognition	2004
**Fesl**	NeuroImage	2003
**Filimon**	NeuroImage	2007
**Floyer-Lea**	Journal of Neurophysiology	2005
**Fox**	Journal of Neurophysiology	1985
**Fox**	Human Brain Mapping	2004
**Furlong**	NeuroImage	2004
**Gavazzi**	Journal of Computer Assisted Tomography	2007
**Gelnar**	NeuroImage	1999
**Gerardin**	Cerebral Cortex	2000
**Gerardin**	Cerebral Cortex	2003
**Gizewski**	NeuroImage	2007
**Gosain**	Plastic and Reconstructive Surgery	2001
**Grafton**	Experimental Brain Research	1993
**Guillot**	Human Brain Mapping	2008
**Hanakawa**	Cerebral Cortex	2008
**Hanakawa**	Journal of Neurophysiology	2003

**Hesselmann**	Brain Topography	2004
**Jancke**	Neuropsychologia	1998
**Jancke**	NeuroImage	1999
**Jancke**	Neuropsychologia	2000
**Joliot**	NeuroImage	1999
**Joliot**	NeuroImage	1998
**Jueptner**	NeuroImage	1997
**Katanoda**	Human Brain Mapping	2001
**Kawashima**	Neuroscience	1999
**Kitada**	NeuroImage	2005
**Kozel**	Journal of Neuropsychiatry and Clinical Neuroscience	2004
**Kroliczak**	Journal of Neurophysiology	2007
**Kuhtz-Buschbeck**	Journal of Urology	2005
**Kuhtz-Buschbeck**	European Journal of Neuroscience	2003
**Langheim**	NeuroImage	2002
**Lee**	Cerebral Cortex	2008
**Lehericy**	Cerebral Cortex	2006
**Lerner**	NeuroImage	2004
**Lotze**	Neuroreport	2000
**Mainero**	NeuroImage	2004
**Maldjian**	NeuroImage	1998
**Mallol**	Brain Research	2007
**Martin**	Journal of Neurophysiology	2004
**Mattay**	Psychiatry Research	1998
**Mayer**	Neuroreport	2001
**Mazoyer**	Brain Research Bulletin	2001
**Milner**	NeuroImage	2007
**Mitchell**	British Journal of Psychiatry	2004
**Mitchell**	Neuropsychologia	2003
**Moore**	Journal of Neurophysiology	2000

**Mostofsky**	Biological Psychiatry	2006
**Muley**	NeuroImage	2001
**Muller**	American Journal of Psychiatry	2003
**Muller**	Progress In Neuro-Psychopharmacology & Biological Psychiatry	2002
**Nakamura**	Brain	2000
**Nour**	Brain	2000
**Omori**	Neuroscience Research	1999
**Onozuka**	Journal of Dental Research	2002
**Onozuka**	Journal of Dental Research	2003
**Puttemans**	Journal of Neuroscience	2005
**Ragland**	American Journal of Psychiatry	2001
**Ramsey**	Journal of Cerebral Blood Flow and Metabolism	1996
**Riecker**	Brain and Language	2000
**Riecker**	NeuroImage	2006
**Rotte**	Stereotactic and Functional Neurosurgery	2002
**Rounis**	NeuroImage	2005
**Sadato**	Brain	1998
**Sadato**	Journal of Neuroscience	1996
**Sadato**	Journal of Neuroscience	1997
**Salgado-Pineda**	NeuroImage	2004
**Seitz**	European Journal of Neuroscience	1992
**Seitz**	Experimental Brain Research	2000
**Seitz**	Stroke	1999
**Seseke**	NeuroImage	2006
**Seseke**	NeuroImage	2008
**Soros**	NeuroImage	2006
**Stephan**	NeuroImage	2002
**Stern**	Brain Research	2007
**Stevens**	Archives of General Psychiatry	1998
**Takada**	Neuroscience Letters	2004

**Thaut**	PLoS ONE	2008
**Umetsu**	NeuroImage	2002
**Van**	Proceedings of the National Academy of Sciences	2005
**Warburton**	Brain	1996
**Watanabe**	NeuroImage	2004
**Wild**	Psychiatry Research	2003
**Wilson**	Nature Neuroscience	2004
**Wise**	Brain	1991
**Yin**	Journal of Nuclear Medicine	2006
**Yokoyama**	Neuroscience	2007
**Yoo**	International Journal of Neuroscience	2005
**Zhang**	NeuroImage	2005

TAB S7 PAPERS INCLUDED IN THE METAANALYSIS INVOLVING REWARD

1st Auth.	Journal	Year
**Berns**	Journal of Neuroscience	2001
**Bjork**	Journal of Neuroscience	2004
**Bjork**	Behavioural Brain Research	2007
**Dillon**	Psychophysiology	2007
**Elliott**	Journal of Neuroscience	2003
**Ernst**	NeuroImage	2005
**Haruno**	Journal of Neuroscience	2004
**Keedwell**	Biological Psychiatry	2005
**Knutson**	Journal of Neuroscience	2005
**Knutson**	Journal of Neuroscience	2001
**Knutson**	NeuroImage	2000
**Knutson**	NeuroImage	2003
**Knutson**	Neuroreport	2001
**Liu**	Journal of Neuroscience	2007
**Martin-Soelch**	European Journal of Neuroscience	2001

**Martin-Soelch**	European Journal of Neuroscience	2003
**Martin-Solch**	Experimental Brain Research	2001
**O'Doherty**	Neuropsychologia	2003
**Ramnani**	NeuroImage	2004
**Rilling**	Neuroreport	2004
**Scheres**	Biological Psychiatry	2007
**Strohle**	NeuroImage	2008
**Ullsperger**	Journal of Neuroscience	2003
**Vartanian**	Neuroreport	2004
**Winston**	Neuropsychologia	2007

TAB S8 PAPERS INCLUDED IN THE METAANALYSIS INVOLVING TACTILE STIMULATION

1st Auth.	Journal	Year
**Aziz**	Journal of Neuroscience	2000
**Binkofski**	Experimental Brain Research	1999
**Binkofski**	European Journal of Neuroscience	1999
**Blakemore**	NeuroImage	1999
**Blaxton**	Journal of Neuroscience	1996
**Bodegard**	Neuron	2001
**Bodegard**	Neuroreport	2000
**Burton**	Cerebral Cortex	1997
**Burton**	Cerebral Cortex	1999
**Bushara**	Neuroreport	2001
**Carlsson**	Journal of Cognitive Neuroscience	2000
**Cheng**	Current Biology	2007
**Coan**	Psychological Science	2006
**Eickhoff**	NeuroImage	2006
**Francis**	Neuroreport	1999
**Giesecke**	Arthritis & Rheumatism	2004
**Hagen**	European Journal of Neuroscience	2002

**Herath**	Cerebral Cortex	2001
**Hlushchuk**	Journal of Neuroscience	2006
**Hobday**	Brain	2001
**Hui**	NeuroImage	2005
**Iadarola**	Brain	1998
**Johansen-Berg**	Neuroreport	2000
**Kitada**	Neuroreport	2003
**Kitada**	NeuroImage	2005
**Kulkami**	European Journal of Neuroscience	2005
**Kumari**	Psychiatry Research	2003
**Lepage**	NeuroImage	2001
**Lloyd**	Nature Neuroscience	2003
**Lotze**	NeuroImage	2001
**Lowell**	NeuroImage	2008
**Maihofner**	European Journal of Neuroscience	2007
**Mehnert**	NeuroImage	2008
**Merabet**	PLoS ONE	2008
**Moore**	Journal of Neurophysiology	2000
**Naito**	Journal of Neurophysiology	2000
**Napadow**	Human Brain Mapping	2005
**Numminen**	NeuroImage	2004
**Paus**	Journal of Neurophysiology	1993
**Rolls**	Cerebral Cortex	2003
**Sadato**	Brain	1998
**Sadato**	Nature	1996
**Sadato**	NeuroImage	2002
**Seitz**	European Journal of Neuroscience	1991
**Stoesz**	International Journal of Psychophysiology	2003
**Van**	Proceedings of the National Academy of Sciences	2005
**Weder**	Human Brain Mapping	2000
**Yoo**	Neuroreport	2003
**Yoo**	NeuroImage	2004

TAB S9 ALE CLUSTERS RELATIVE TO THE METAANALYSIS OF ATTENTIONAL TASKS

Cluster #	Volume (mm^3)	Weighted Center (x,y,z)			x	y	z	Label
1	254744	-1.89	-19.45	25.6	-2	2	50	Medial Frontal Gyrus Brodmann area 6
					32	20	4	Sub-lobar Insula Brodmann area 13
					-42	4	32	Precentral Gyrus Brodmann area 9
					-26	-64	42	Superior Parietal Lobule Brodmann area 7
					44	8	32	Middle Frontal Gyrus Brodmann area 9
					-32	18	8	Sub-lobar Insula Brodmann area 13
					28	-62	42	Superior Parietal Lobule Brodmann area 7
					-36	-48	40	Inferior Parietal Lobule Brodmann area 40
					22	-64	48	Precuneus Brodmann area 7
					-30	-12	50	Precentral Gyrus Brodmann area 6
					-38	24	28	Middle Frontal Gyrus Brodmann area 9
					6	-18	6	Sub-lobar Thalamus Medial Dorsal Nucleus
					-12	-16	8	Sub-lobar Thalamus *
					-36	-24	50	Precentral Gyrus Brodmann area 4
					-40	-70	-2	Inferior Occipital Gyrus Brodmann area 19
					-36	-66	-12	Posterior Lobe Declive *
					44	-64	-4	Inferior Temporal Gyrus Brodmann area 37
					28	-74	28	Precuneus Brodmann area 19
					24	-10	52	Middle Frontal Gyrus Brodmann area 6
					12	-6	14	Sub-lobar Thalamus Ventral Anterior Nucleus
					36	34	30	Superior Frontal Gyrus Brodmann area 9
					-28	-82	16	Middle Occipital Gyrus Brodmann area 19
					-26	-78	22	Middle Occipital Gyrus Brodmann area 19
					54	-40	14	Superior Temporal Gyrus Brodmann area 13
					-36	-56	-22	Anterior Lobe Culmen *

					0	32	20	Anterior Cingulate Brodmann area 32
					48	-44	34	Supramarginal Gyrus Brodmann area 40
					-30	-84	-2	Middle Occipital Gyrus Brodmann area 18
					-16	2	8	Sub-lobar Lentiform Nucleus Putamen
					36	-56	-16	Posterior Lobe Declive *
					30	-80	16	Middle Occipital Gyrus Brodmann area 19
					-52	-28	10	Superior Temporal Gyrus Brodmann area 41
					18	6	0	Sub-lobar Lentiform Nucleus Putamen
					-42	-2	4	Sub-lobar Insula Brodmann area 13
					32	44	22	Middle Frontal Gyrus Brodmann area 10
					-52	-30	22	Inferior Parietal Lobule Brodmann area 40
					-10	-64	-4	Anterior Lobe Culmen *
					-52	-44	6	Middle Temporal Gyrus Brodmann area 21
					14	-88	6	Lingual Gyrus Brodmann area 17
					24	-48	-22	Anterior Lobe Culmen *
					-2	-84	8	Cuneus Brodmann area 17
					22	-4	-14	Parahippocampal Gyrus Amygdala
					-6	-82	-2	Lingual Gyrus Brodmann area 18
					36	-22	50	Precentral Gyrus Brodmann area 4
					10	-80	0	Lingual Gyrus Brodmann area 18
					54	-34	34	Inferior Parietal Lobule Brodmann area 40
					-22	-76	-16	Posterior Lobe Declive *
					20	-64	-12	Posterior Lobe Declive *
					-16	-84	0	Lingual Gyrus Brodmann area 17
					-22	-58	-8	Fusiform Gyrus Brodmann area 19
					-18	-28	-2	Sub-lobar Thalamus *
					-20	-92	-10	Inferior Occipital Gyrus Brodmann area 17
					56	-20	6	Superior Temporal Gyrus Brodmann area 41
					-44	40	6	Inferior Frontal Gyrus Brodmann area 46
**2**	680	4.58	-47.98	28.29	6	-48	28	Cingulate Gyrus Brodmann area 31
**3**	616	-24.75	-4.05	-14.73	-24	-4	-14	Parahippocampal Gyrus Amygdala
**4**	432	-30.87	50.02	10.46	-32	50	10	Middle Frontal Gyrus Brodmann area 10
**5**	424	20.04	-30.01	0	20	-30	-2	Parahippocampal Gyrus Brodmann area 27

**6**	216	32.14	52.17	7.91	32	52	8	Middle Frontal Gyrus Brodmann area 10
**7**	104	44.16	-16.92	14.15	44	-16	14	Sub-lobar Insula Brodmann area 13

ALE MAPS WERE COMPUTED AT AN FDR-CORRECTED THRESHOLD OF P < 0.05; MINIMUM CLUSTER DIMENSION K>100MM3

TAB S10 ALE CLUSTERS RELATIVE TO THE METAANALYSIS OF PAIN-RELATED TASKS

**Cluster #**	**Volume (mm^3)**	**Weighted.Center.(x,y,z)**			**x**	**y**	**z**	**Label**
**1**	94616	1.43	-8.29	12.58	34	10	8	Sub-lobar Insula Brodmann area 13
					-52	-26	20	Postcentral Gyrus Brodmann area 40
					-32	8	8	Sub-lobar Claustrum *
					-12	-16	8	Sub-lobar Thalamus *
					48	-26	20	Sub-lobar Insula Brodmann area 13
					8	-16	8	Sub-lobar Thalamus Medial Dorsal Nucleus
					46	-48	44	Inferior Parietal Lobule Brodmann area 40
					50	4	12	Inferior Frontal Gyrus Brodmann area 44
					-40	-22	18	Sub-lobar Insula Brodmann area 13
					36	-20	16	Sub-lobar Insula Brodmann area 13
					38	-32	50	Postcentral Gyrus Brodmann area 3
					2	-24	-2	Right Brainstem Midbrain * Red Nucleus
					-30	-14	10	Sub-lobar Lentiform Nucleus Putamen
					42	2	40	Middle Frontal Gyrus Brodmann area 6
					18	6	2	Sub-lobar Lentiform Nucleus Putamen
					48	-30	44	Inferior Parietal Lobule Brodmann area 40
					50	4	30	Inferior Frontal Gyrus Brodmann area 9
					-58	-28	34	Inferior Parietal Lobule Brodmann area 40
					36	-54	50	Superior Parietal Lobule Brodmann area 7
					58	-38	32	Supramarginal Gyrus Brodmann area 40
					56	-24	36	Postcentral Gyrus Brodmann area 2
**2**	26624	-0.77	7.75	38.35	2	0	44	Cingulate Gyrus Brodmann area 24
					-4	18	36	Cingulate Gyrus Brodmann area 32
					8	20	26	Cingulate Gyrus Brodmann area 32
					-6	36	26	Anterior Cingulate Brodmann area 32
					-10	-8	58	Medial Frontal Gyrus Brodmann area 6

**3**	8976	33.72	42.04	13.96	36	38	28	Superior Frontal Gyrus Brodmann area 9
					38	44	2	Inferior Frontal Gyrus *
					20	48	8	Medial Frontal Gyrus Brodmann area 10
**4**	4544	-33.21	-41.92	52.31	-28	-40	54	Sub-Gyral Brodmann area 40
					-40	-48	50	Inferior Parietal Lobule Brodmann area 40
					-28	-58	44	Superior Parietal Lobule Brodmann area 7
					-36	-54	36	Inferior Parietal Lobule Brodmann area 40
					-20	-40	68	Postcentral Gyrus Brodmann area 5
**5**	3904	-29.77	-54.76	-27.16	-30	-58	-26	Anterior Lobe Culmen *
**6**	2912	24.23	-58.67	-22.68	24	-60	-22	Posterior Lobe Declive *
**7**	1880	0.89	-51.17	-14.95	2	-48	-16	Anterior Lobe Cerebellar Lingual *
**8**	1248	-37.2	36.56	20.29	-40	32	18	Middle Frontal Gyrus Brodmann area 46
					-36	38	22	Middle Frontal Gyrus Brodmann area 10
**9**	864	0.12	-42.41	23.45	0	-40	24	Posterior Cingulate Brodmann area 23
**10**	840	-23.59	-16.71	54.28	-24	-16	54	Precentral Gyrus Brodmann area 6
**11**	744	-6.16	-36.64	-25.94	-6	-36	-26	Anterior Lobe * *
**12**	360	4.22	47.37	6.92	2	52	6	Medial Frontal Gyrus Brodmann area 10
					6	42	8	Anterior Cingulate Brodmann area 32
**13**	264	4.9	-60.93	-26.06	4	-62	-26	Anterior Lobe Nodule *
**14**	192	-32.51	43.32	1.91	-32	44	2	Sub-Gyral *
**15**	192	46.75	-17.08	42.07	46	-16	42	Precentral Gyrus Brodmann area 4

ALE MAPS WERE COMPUTED AT AN FDR-CORRECTED THRESHOLD OF P < 0.05; MINIMUM CLUSTER DIMENSION K>100MM3

TAB S11 ALE CLUSTERS RELATIVE TO THE METAANALYSIS OF EMOTIONAL TASKS

Cluster #	Volume (mm^3)	Weighted Center (x,y,z)			x	y	z	Label
**1**	64128	1.43	-5.04	0.4	0	-14	10	Sub-lobar Thalamus Medial Dorsal Nucleus
					4	-2	-4	Sub-lobar * Hypothalamus
					-20	-6	-14	Parahippocampal Gyrus Amygdala
					-34	16	6	Sub-lobar Insula Brodmann area 13
					-40	-4	8	Sub-lobar Insula Brodmann area 13
					4	-26	-8	Right Brainstem Midbrain * Red Nucleus
					36	18	10	Sub-lobar Insula Brodmann area 13
					40	-2	6	Sub-lobar Insula Brodmann area 13
					28	8	4	Sub-lobar Lentiform Nucleus Putamen

					20	-6	-12	Parahippocampal Gyrus Amygdala
					-10	0	6	Sub-lobar Lentiform Nucleus *
					-26	-26	-4	Sub-Gyral Hippocampus
					-24	0	10	Sub-lobar Lentiform Nucleus Putamen
					28	-18	-2	Sub-lobar Lentiform Nucleus Lateral Globus Pallidus
					32	-12	12	Sub-lobar Claustrum *
					-4	-48	-14	Anterior Lobe Cerebellar Lingual *
					-14	16	-4	Sub-lobar Caudate Caudate Head
					46	22	-2	Inferior Frontal Gyrus Brodmann area 47
					48	24	6	Inferior Frontal Gyrus Brodmann area 45
					32	-26	-6	Sub-Gyral Hippocampus
					32	0	16	Sub-lobar Insula Brodmann area 13
					4	-24	-30	No Gray Matter found
					54	6	12	Inferior Frontal Gyrus Brodmann area 44
					48	12	20	Inferior Frontal Gyrus Brodmann area 44
					0	8	16	Sub-lobar Caudate Caudate Body
**2**	9416	-0.33	7	36.43	-4	-8	38	Cingulate Gyrus Brodmann area 24
					6	16	32	Cingulate Gyrus Brodmann area 32
					2	14	30	Cingulate Gyrus Brodmann area 24
					0	22	38	Cingulate Gyrus Brodmann area 32
					-2	4	42	Cingulate Gyrus Brodmann area 24
**3**	4360	42.14	-60.2	-10.34	44	-62	-8	Fusiform Gyrus Brodmann area 37
					34	-52	-18	Anterior Lobe Culmen *
**4**	3712	-42.72	-63.33	-11.18	-46	-66	-8	Middle Occipital Gyrus Brodmann area 37
**5**	2672	-0.41	26.99	-4.61	2	24	-6	Anterior Cingulate Brodmann area 24
					-4	40	4	Anterior Cingulate Brodmann area 32
**6**	1560	-51.12	-27.5	20.6	-52	-30	22	Inferior Parietal Lobule Brodmann area 40
**7**	1464	26.77	-80.45	-4.94	26	-80	-6	Lingual Gyrus Brodmann area 18
					24	-86	4	Middle Occipital Gyrus Brodmann area 19
**8**	1200	52.81	-29.07	22.4	54	-32	24	Inferior Parietal Lobule Brodmann area 40
					50	-20	18	Sub-lobar Insula Brodmann area 40
**9**	848	-26.05	-44.58	-15.05	-24	-44	-16	Anterior Lobe Culmen *
**10**	696	0.54	48.14	-3.73	0	48	-4	Anterior Cingulate Brodmann area 32
**11**	640	-11.13	-27.19	63.26	-12	-26	64	Precentral Gyrus Brodmann area 4
					-4	-30	62	Paracentral Lobule Brodmann area 6

**12**	600	-25.79	-84.93	-3.07	-26	-84	-4	Middle Occipital Gyrus Brodmann area 18
**13**	552	45.39	1.89	30.32	46	2	30	Inferior Frontal Gyrus Brodmann area 6
**14**	432	-47.01	3.6	30.13	-46	2	30	Inferior Frontal Gyrus Brodmann area 6
**15**	424	0.47	30.99	17.36	0	32	16	Anterior Cingulate Brodmann area 24
**16**	368	-46.74	-32.83	36.36	-46	-32	36	Inferior Parietal Lobule Brodmann area 40
**17**	336	17.71	-28.15	59.51	18	-28	60	Precentral Gyrus Brodmann area 4
**18**	232	-43.33	26.57	11.87	-44	26	12	Inferior Frontal Gyrus Brodmann area 13
**19**	208	-18.61	54.47	24.31	-18	54	24	Superior Frontal Gyrus Brodmann area 10
**20**	112	1.14	-49.85	24.57	2	-50	24	Posterior Cingulate Brodmann area 23

ALE MAPS WERE COMPUTED AT AN FDR-CORRECTED THRESHOLD OF P < 0.05; MINIMUM CLUSTER DIMENSION K>100MM3

TAB S12 ALE CLUSTERS RELATIVE TO THE METAANALYSIS OF REWARD TASKS

Cluster #	Volume (mm^3)	Weighted Center (x,y,z)			x	y	z	Label
1	8064	-20.29	-2.39	-10.15	-22	-6	-12	Parahippocampal Gyrus Amygdala
					-18	10	-2	Sub-lobar Lentiform Nucleus Putamen
					-4	-4	0	Sub-lobar Thalamus *
					-10	2	0	Sub-lobar Lentiform Nucleus Medial Globus Pallidus
					-22	4	8	Sub-lobar Lentiform Nucleus Putamen
2	5120	21.95	-6.75	-10.81	26	-4	-12	Parahippocampal Gyrus Amygdala
					18	2	-10	Sub-lobar Lentiform Nucleus Putamen
					16	-28	-12	Anterior Lobe Culmen *
					20	-18	-8	Parahippocampal Gyrus Brodmann area 35
					12	-16	-2	Right Brainstem Midbrain * Subthalamic Nucleus
3	3144	-39.96	-52.66	-17.93	-38	-56	-18	Posterior Lobe Declive *
					-44	-50	-16	Fusiform Gyrus Brodmann area 37
					-36	-44	-20	Anterior Lobe Culmen *
4	2440	-44.66	9.2	-14.69	-30	20	-8	Inferior Frontal Gyrus Brodmann area 47
					-52	6	-18	Middle Temporal Gyrus Brodmann area 21
					-52	-4	-14	Middle Temporal Gyrus Brodmann area 21
					-48	12	-18	Superior Temporal Gyrus Brodmann area 38

					-36	12	-20	Superior Temporal Gyrus Brodmann area 38
5	2368	34.2	-77.02	-12.81	40	-74	-8	Inferior Occipital Gyrus Brodmann area 19
					32	-80	-16	Posterior Lobe Declive *
6	2224	-55.62	-21.42	2.73	-54	-18	4	Superior Temporal Gyrus Brodmann area 41
					-60	-34	-4	Middle Temporal Gyrus Brodmann area 21
					-58	-28	6	Superior Temporal Gyrus Brodmann area 22
7	1136	1.01	49.42	21.17	2	48	22	Medial Frontal Gyrus Brodmann area 9
8	1048	-44.64	17.58	-1.28	-46	14	0	Inferior Frontal Gyrus Brodmann area 47
					-48	22	-2	Inferior Frontal Gyrus Brodmann area 47
9	992	43.73	19.79	-6.81	42	16	-10	Inferior Frontal Gyrus Brodmann area 47
					42	24	-4	Inferior Frontal Gyrus Brodmann area 47
10	688	55.91	-31.32	22.8	58	-30	22	Inferior Parietal Lobule Brodmann area 40
11	648	-1.45	-23.51	-5.76	-2	-24	-6	Left Brainstem Midbrain * Red Nucleus
12	616	53.33	6.43	12.5	54	6	12	Inferior Frontal Gyrus Brodmann area 44
13	584	24.32	-57.96	-15.8	26	-56	-18	Posterior Lobe Declive *
					22	-60	-12	Posterior Lobe Declive *
14	568	-31.81	-24.02	-14.38	-32	-24	-14	Parahippocampal Gyrus Hippocampus
15	504	21.99	0.72	6.67	22	0	6	Sub-lobar Lentiform Nucleus Putamen
16	448	0.03	39.36	7.4	0	38	8	Anterior Cingulate Brodmann area 24
17	448	37.16	37.64	16.25	38	38	16	Middle Frontal Gyrus Brodmann area 10
18	416	-52.33	-62.36	3.42	-52	-66	4	Middle Temporal Gyrus Brodmann area 37
					-54	-60	2	Middle Temporal Gyrus Brodmann area 37
19	416	-2.18	-6.57	52.38	-2	-8	52	Medial Frontal Gyrus Brodmann area 6
20	400	10.99	19.95	0.03	10	20	0	Sub-lobar Caudate Caudate Head
21	376	-27.69	-48.86	6.46	-28	-48	6	Parahippocampal Gyrus Brodmann area 30
22	344	-33.77	-79.52	-14.26	-34	-80	-14	Fusiform Gyrus Brodmann area 19
23	344	-5.23	28.96	-0.12	-6	28	0	Anterior Cingulate Brodmann area 24
24	296	-41.24	-70.68	15.45	-42	-70	16	Middle Temporal Gyrus Brodmann area 39
25	288	49.41	-54.09	1.01	48	-54	2	Middle Temporal Gyrus Brodmann area 37
26	280	-5.9	16.23	58.99	-6	16	58	Superior Frontal Gyrus Brodmann area 6
27	248	50.79	2.91	41.66	50	2	42	Middle Frontal Gyrus Brodmann area 6

28	240	55.81	-22.4	5.87	56	-22	6	Superior Temporal Gyrus Brodmann area 41
29	208	-21.37	-73.06	-13.84	-20	-74	-12	Posterior Lobe Declive *
30	200	-23.68	16.96	49.99	-24	18	50	Middle Frontal Gyrus Brodmann area 6
31	192	45.15	-4.52	-14.34	44	-6	-16	Sub-Gyral Brodmann area 20
					46	-2	-14	Superior Temporal Gyrus Brodmann area 21
32	184	48.61	-37.57	5.65	48	-38	6	Middle Temporal Gyrus Brodmann area 22
33	160	19	64	12	18	64	12	Superior Frontal Gyrus Brodmann area 10
34	160	15	-39	68	16	-40	68	Postcentral Gyrus Brodmann area 3
35	152	-15.71	-1.68	21.28	-16	-2	22	Sub-lobar Caudate Caudate Body
36	136	-21.64	-96.12	2.11	-22	-96	2	Middle Occipital Gyrus Brodmann area 18
37	136	49.42	16.82	23.99	50	16	24	Inferior Frontal Gyrus Brodmann area 9
38	128	-17.24	-13.59	28.14	-18	-14	28	Sub-lobar Caudate Caudate Body
39	120	-51.84	-45.06	6.17	-52	-44	6	Middle Temporal Gyrus Brodmann area 21
40	120	-31.6	-19.74	45.21	-32	-20	46	Postcentral Gyrus Brodmann area 3
41	112	-12.29	29.99	48.99	-12	30	48	Superior Frontal Gyrus Brodmann area 8

ALE MAPS WERE COMPUTED AT AN FDR-CORRECTED THRESHOLD OF $P < 0.05$; MINIMUM CLUSTER DIMENSION K>100MM^3

TAB S13 ALE CLUSTERS RELATIVE TO THE METAANALYSIS OF INTEROCEPTION TASKS

Cluster #	Volume (mm^3)	Weighted Center (x,y,z)			x	y	z	Label
**1**	60024	2.27	-5.25	0.03	0	-14	10	Sub-lobar Thalamus Medial Dorsal Nucleus
					4	-2	-4	Sub-lobar * Hypothalamus
					-20	-4	-14	Parahippocampal Gyrus Amygdala
					-34	16	6	Sub-lobar Insula Brodmann area 13
					-40	-4	8	Sub-lobar Insula Brodmann area 13
					4	-26	-8	Right Brainstem Midbrain * Red Nucleus
					38	-2	6	Sub-lobar Claustrum *
					36	16	10	Sub-lobar Insula Brodmann area 13
					-26	18	2	Sub-lobar Claustrum *
					20	-6	-12	Parahippocampal Gyrus Amygdala
					28	6	2	Sub-lobar Lentiform Nucleus Putamen
					-12	-2	4	Sub-lobar Lentiform Nucleus *
					-28	-22	0	Sub-lobar Lentiform Nucleus Putamen
					28	-18	-2	Sub-lobar Lentiform Nucleus Lateral

								Globus Pallidus
					32	-12	12	Sub-lobar Claustrum *
					-32	4	16	Sub-lobar Insula Brodmann area 13
					-4	-48	-14	Anterior Lobe Cerebellar Lingual *
					-24	0	12	Sub-lobar Lentiform Nucleus Putamen
					48	24	6	Inferior Frontal Gyrus Brodmann area 45
					32	-26	-6	Sub-Gyral Hippocampus
					-18	-24	-6	Left Brainstem Midbrain Thalamus Medial Geniculum Body
					4	-24	-30	No Gray Matter found
					54	6	12	Inferior Frontal Gyrus Brodmann area 44
					46	22	-2	Inferior Frontal Gyrus Brodmann area 47
					48	12	20	Inferior Frontal Gyrus Brodmann area 44
					-16	18	-4	Sub-lobar Lentiform Nucleus Putamen
**2**	9528	-0.85	7.48	35.81	-6	-8	38	Cingulate Gyrus Brodmann area 24
					0	14	28	Cingulate Gyrus Brodmann area 24
					0	22	38	Cingulate Gyrus Brodmann area 32
					2	8	16	Sub-lobar Caudate Caudate Body
**3**	4088	42.46	-60.25	-10.23	44	-62	-8	Fusiform Gyrus Brodmann area 37
					32	-50	-18	Anterior Lobe Culmen *
**4**	3344	-43.24	-64.42	-10.32	-46	-66	-8	Middle Occipital Gyrus Brodmann area 37
**5**	2520	-0.24	26.84	-4.07	2	24	-6	Anterior Cingulate Brodmann area 24
					-6	38	4	Anterior Cingulate Brodmann area 24
					-6	34	-4	Anterior Cingulate Brodmann area 32
**6**	1720	-51.25	-27.65	20.88	-52	-30	22	Inferior Parietal Lobule Brodmann area 40
**7**	1568	52.8	-28.35	22.18	54	-32	24	Inferior Parietal Lobule Brodmann area 40
					50	-20	18	Sub-lobar Insula Brodmann area 40
**8**	1336	26.82	-80.66	-4.77	28	-80	-6	Middle Occipital Gyrus Brodmann area 18
					24	-86	2	Middle Occipital Gyrus Brodmann area 18
**9**	664	-12.84	-26.8	63.39	-12	-26	64	Precentral Gyrus Brodmann area 4
**10**	648	-25.64	-84.6	-3.76	-26	-84	-4	Middle Occipital Gyrus Brodmann area 18
**11**	608	0.56	30.97	17.48	0	32	16	Anterior Cingulate Brodmann area 24
**12**	592	0.8	47.44	-4.22	2	48	-6	Medial Frontal Gyrus Brodmann area 10
**13**	480	-26.9	-44.46	-14.94	-26	-44	-16	Anterior Lobe Culmen *
**14**	408	-46.15	3.05	30.21	-46	2	30	Inferior Frontal Gyrus Brodmann area 6
**15**	224	-18.48	54.46	24.21	-18	54	24	Superior Frontal Gyrus Brodmann area 10
**16**	216	45.75	1.63	30.21	46	2	30	Inferior Frontal Gyrus Brodmann area 6
**17**	184	1.13	-49.5	24.85	2	-50	24	Posterior Cingulate Brodmann area 23

**18**	168	-46.38	-31.82	36.4	-46	-32	36	Inferior Parietal Lobule Brodmann area 40
**19**	160	-39.29	40.7	17.8	-38	40	18	Middle Frontal Gyrus Brodmann area 10
**20**	112	15.14	32.57	15.14	16	32	14	Anterior Cingulate Brodmann area 32

ALE MAPS WERE COMPUTED AT AN FDR-CORRECTED THRESHOLD OF P < 0.05; MINIMUM CLUSTER DIMENSION K>100MM3

TAB S14 ALE CLUSTERS RELATIVE TO THE METAANALYSIS OF MEMORY TASKS

Cluster #	Volume (mm^3)	Weighted Center (x,y,z)			x	y	z	Label
**1**	2576	-11.05	-71.78	32.17	-26	-74	30	Left Precuneus Brodmann area 19
					2	-70	28	Right Precuneus Brodmann area 31
					-8	-70	28	Left Precuneus Brodmann area 31
					6	-70	44	Right Precuneus Brodmann area 7
**2**	1960	-39.3	6.61	33.57	-38	6	32	Left Precentral Gyrus Brodmann area 9
**3**	1448	-30.06	20.1	5.4	-30	22	8	Left Insula Brodmann area 13
					-36	14	-4	Left Insula Brodmann area 13
**4**	1440	-8.12	-80.84	-8.43	-8	-82	-6	Left Lingual Gyrus Brodmann area 18
					-6	-74	-12	Left Declive
**5**	1136	19.55	50.67	3.89	14	52	0	Right Medial Frontal Gyrus Brodmann area 10
					26	52	6	Right Superior Frontal Gyrus Brodmann area 10
**6**	1128	-30.34	-55.18	42.51	-26	-56	42	Left Superior Parietal Lobule Brodmann area 7
					-42	-54	44	Left Inferior Parietal Lobule Brodmann area 40
**7**	1024	-0.04	-49.12	39.05	0	-50	42	Left Precuneus Brodmann area 7
					2	-48	28	Right Cingulate Gyrus Brodmann area 31
**8**	952	-0.87	-8.74	59.5	0	-10	60	Left Medial Frontal Gyrus Brodmann area 6
**9**	848	35.83	-57.15	44.73	36	-56	44	Right Inferior Parietal Lobule Brodmann area 40
**10**	800	-32.12	43.55	17.34	-32	42	20	Left Middle Frontal Gyrus Brodmann area 10
					-32	46	12	Left Middle Frontal Gyrus Brodmann area 10
**11**	752	8.26	-74.7	-3.8	8	-74	-4	Right Lingual Gyrus Brodmann area 18
**12**	728	-44.35	37.08	-5.65	-44	38	-6	Left Middle Frontal Gyrus Brodmann area 47
**13**	720	-2.39	12.12	45.72	-2	12	44	Left Medial Frontal Gyrus Brodmann area 6

**14**	664	18.77	-75.26	-33.65	18	-76	-34	Right Pyramis
**15**	664	-4.82	45.9	6.46	-6	46	6	Left Anterior Cingulate Brodmann area 32
**16**	632	29.76	23.25	8.02	30	24	8	Right Insula Brodmann area 13
**17**	608	-17.72	-28.31	-8.02	-18	-28	-8	Left Parahippocampal Gyrus Brodmann area 28
**18**	536	-21.44	-76.5	-34.62	-22	-76	-34	Left Pyramis
**19**	512	21.23	-76.72	36.43	22	-78	36	Right Precuneus Brodmann area 19
**20**	496	17.17	-28.47	-7.23	18	-28	-8	Right Parahippocampal Gyrus Brodmann area 35
**21**	472	6.98	-18.81	8.4	8	-18	8	Right Medial Dorsal Nucleus
**22**	448	-29.49	-29.91	51.95	-30	-30	52	Left Precentral Gyrus Brodmann area 4
**23**	400	-26.84	-90	-0.89	-26	-90	0	Left inferior Occipital Gyrus Brodmann area 18
**24**	320	12.84	7.47	53.13	16	6	54	Right Medial Frontal Gyrus Brodmann area 6
					8	10	52	Right Superior Frontal Gyrus Brodmann area 6
**25**	208	-3.28	37.91	23.85	-4	38	24	Left Anterior Cingulate Brodmann area 32
**26**	208	-49.76	-40.25	27.15	-50	-40	28	Left Inferior Parietal Lobule Brodmann area 40
**27**	200	-40.15	-15.28	-23.82	-40	-16	-24	Left Fusiform Gyrus Brodmann area 20
**28**	160	32.09	12.09	-7.9	32	12	-8	Right insula Brodmann area 13
**29**	160	-19.91	3.91	-3.9	-20	4	-4	Left Putamen
**30**	152	0.56	-38.02	-16	0	-38	-16	Left Cerebellar Lingual
**31**	152	-39.99	-59.99	11.99	-40	-60	12	Left Middle Temporal Gyrus Brodmann area 19
**32**	152	-50.01	-35.99	12.02	-50	-36	12	Left Superior Temporal Gyrus Brodmann area 41
**33**	152	37.99	42.01	12.01	38	42	12	Right Middle Frontal Gyrus Brodmann area 10
**34**	144	41.88	-64	-0.13	42	-66	0	Right Inferior Temporal Gyrus
**35**	136	35.88	29.41	35.78	36	30	36	Right Middle Frontal Gyrus Brodmann area 9
**36**	128	-14	31.88	-19.65	-14	32	-20	Left Inferior Frontal Gyrus Brodmann area 11
**37**	128	-15.5	1.96	7.02	-16	2	8	Left Putamen
**38**	120	-40.81	-18.55	46.04	-40	-18	46	Left Postcentral Gyrus Brodmann area 3
**39**	112	-53.47	-10.13	-11.87	-54	-10	-12	Left Middle Temporal Gyrus Brodmann area 21

ALE MAPS WERE COMPUTED AT AN FDR-CORRECTED THRESHOLD OF P < 0.05; MINIMUM CLUSTER DIMENSION K>100MM3

TAB S15 ALE CLUSTERS RELATIVE TO THE METAANALYSIS OF MOTOR TASKS

Cluster #	Volume (mm^3)	Weighted Center (x,y,z)			x	y	z	Label
1	106192	-20.6	-14.99	34.72	-4	-8	52	Medial Frontal Gyrus Brodmann area 6
					-38	-28	52	Postcentral Gyrus Brodmann area 3
					-12	-20	6	Sub-lobar Thalamus *
					-22	-6	4	Sub-lobar Lentiform Nucleus Putamen
					-50	-26	16	Postcentral Gyrus Brodmann area 40
					12	-18	6	Sub-lobar Thalamus *
					-52	-2	30	Precentral Gyrus Brodmann area 6
					-38	-42	42	Inferior Parietal Lobule Brodmann area 40
					24	-2	6	Sub-lobar Lentiform Nucleus Putamen
					-50	-16	38	Postcentral Gyrus Brodmann area 3
					-28	-62	52	Superior Parietal Lobule Brodmann area 7
					-24	-68	46	Superior Parietal Lobule Brodmann area 7
					-46	-2	10	Precentral Gyrus Brodmann area 44
					-38	-54	50	Superior Parietal Lobule Brodmann area 7
					-36	-14	18	Sub-lobar Insula Brodmann area 13
					32	18	2	Sub-lobar Insula *
					-48	10	2	Sub-lobar Insula Brodmann area 13
2	38976	42.47	-20.11	38.76	36	-24	54	Precentral Gyrus Brodmann area 4
					38	-42	44	Inferior Parietal Lobule Brodmann area 40
					54	4	24	Inferior Frontal Gyrus Brodmann area 9
					50	-12	34	Precentral Gyrus Brodmann area 6
					56	-24	14	Postcentral Gyrus Brodmann area 40
					54	-28	28	Inferior Parietal Lobule Brodmann area 40
					58	-38	8	Middle Temporal Gyrus Brodmann area 22
					52	-16	2	Superior Temporal Gyrus Brodmann area 22
3	24416	2.85	-53.88	-20.26	16	-50	-20	Anterior Lobe * Dentate
					-20	-54	-22	Anterior Lobe Culmen *
					2	-62	-14	Posterior Lobe Declive *
4	768	-37.13	19.27	4.48	-38	20	4	Sub-lobar Insula Brodmann area 13
5	552	-20.89	-76.06	-17.66	-22	-76	-18	Posterior Lobe Declive *
6	344	-49.1	-42.12	12.67	-48	-44	12	Middle Temporal Gyrus Brodmann area 22

**7**	184	35.46	32.69	32.18	36	32	32	Middle Frontal Gyrus Brodmann area 9
**8**	128	45.77	-55.76	-9.01	46	-56	-10	Fusiform Gyrus Brodmann area 37

ALE MAPS WERE COMPUTED AT AN FDR-CORRECTED THRESHOLD OF P < 0.05; MINIMUM CLUSTER DIMENSION K>100MM3

TAB S16 ALE CLUSTERS RELATIVE TO THE METAANALYSIS OF PERCECTIVE (TACTILE) TASKS

Cluster#	Volume (mm^3)	Weighted Center (x,y,z)			x	y	z	Label
**1**	40224	-43.73	-23.89	30.97	-52	-26	18	Postcentral Gyrus Brodmann area 40
					-40	-38	46	Inferior Parietal Lobule Brodmann area 40
					-38	-26	54	Postcentral Gyrus Brodmann area 3
					-30	-58	48	Superior Parietal Lobule Brodmann area 7
					-48	2	30	Precentral Gyrus Brodmann area 6
					-48	-32	34	Inferior Parietal Lobule Brodmann area 40
					-34	14	4	Sub-lobar Claustrum *
					-36	-12	8	Sub-lobar Claustrum *
					-38	-42	36	Supramarginal Gyrus Brodmann area 40
					-26	-4	4	Sub-lobar Lentiform Nucleus Putamen
**2**	18952	46.31	-32.17	33.04	54	-24	20	Postcentral Gyrus Brodmann area 40
					50	-34	32	Inferior Parietal Lobule Brodmann area 40
					40	-38	42	Inferior Parietal Lobule Brodmann area 40
					36	-54	46	Inferior Parietal Lobule Brodmann area 40
					38	-26	52	Postcentral Gyrus Brodmann area 3
					30	-14	54	Precentral Gyrus Brodmann area 6
					34	-22	40	Postcentral Gyrus Brodmann area 3
					20	-56	52	Precuneus Brodmann area 7
**3**	8200	43.57	9.68	16.34	48	12	12	Inferior Frontal Gyrus Brodmann area 44
					36	18	2	Sub-lobar Insula *
					48	4	26	Inferior Frontal Gyrus Brodmann area 9
					42	2	40	Middle Frontal Gyrus Brodmann area 6
**4**	7728	0.3	1.15	47.92	0	-2	50	Medial Frontal Gyrus Brodmann area 6
**5**	944	19.02	-49.63	-20.43	20	-48	-20	Anterior Lobe Culmen *
**6**	736	-10.83	-15.61	5.11	-10	-14	6	Sub-lobar Thalamus *
**7**	672	32.14	34.47	29.96	32	34	30	Superior Frontal Gyrus Brodmann area 9
**8**	392	0.64	-16.72	-3.66	0	-16	-4	Left Brainstem Midbrain * Red Nucleus

**9**	304	-1.39	-70.4	-23.95	0	-70	-24	Posterior Lobe Tuber of Vermis *
**10**	176	-49.37	6.46	1.82	-50	6	2	Superior Temporal Gyrus Brodmann area 22
**11**	168	-33.91	26.48	33.79	-34	26	34	Middle Frontal Gyrus Brodmann area 9
**12**	136	-38.94	17.89	20.12	-38	18	20	No Gray Matter found

ALE MAPS WERE COMPUTED AT AN FDR-CORRECTED THRESHOLD OF P < 0.05; MINIMUM CLUSTER DIMENSION K>100MM3

TAB S17 "CORE NETWORK" METAANALYTIC CONNECTIVITY

Cluster #	Volume (mm^3)	Weighted Center (x,y,z)			x	y	z	Label
**1**	139208	-2.54	2.91	17.67	30	20	4	Sub-lobar Claustrum *
					-32	18	6	Sub-lobar Insula Brodmann area 13
					-44	4	30	Inferior Frontal Gyrus Brodmann area 9
					12	6	4	Sub-lobar Caudate Caudate Head
					-30	-56	42	Inferior Parietal Lobule Brodmann area 7
					-10	-16	8	Sub-lobar Thalamus Medial Dorsal Nucleus
					40	8	30	Inferior Frontal Gyrus Brodmann area 9
					6	-14	8	Sub-lobar Thalamus Medial Dorsal Nucleus
					40	30	28	Middle Frontal Gyrus Brodmann area 9
					-12	6	4	Sub-lobar Caudate Caudate Head
					-42	26	26	Middle Frontal Gyrus Brodmann area 9
					-40	-46	38	Inferior Parietal Lobule Brodmann area 40
					-28	-8	50	Precentral Gyrus Brodmann area 6
					-38	-6	42	Middle Frontal Gyrus Brodmann area 6
					-52	-34	26	Inferior Parietal Lobule Brodmann area 40
					34	48	18	Middle Frontal Gyrus Brodmann area 10
					-36	46	14	Middle Frontal Gyrus Brodmann area 10
					30	-6	52	Precentral Gyrus Brodmann area 6
					20	-28	0	Sub-lobar Thalamus *
					50	6	12	Precentral Gyrus Brodmann area 44
					-28	-76	22	Middle Occipital Gyrus Brodmann area 19
					-28	-78	18	Middle Occipital Gyrus Brodmann area 19
					-10	-72	38	Precuneus Brodmann area 7
					-36	-2	8	Sub-lobar Claustrum *
**2**	22064	-1.12	10.36	45.33	-4	8	48	Superior Frontal Gyrus Brodmann area 6

**3**	12536	32.13	-54.97	41.31	28	-60	44	Superior Parietal Lobule Brodmann area 7
					30	-70	28	No Gray Matter found
**4**	6528	-39.38	-58.49	-15.85	-38	-56	-18	Posterior Lobe Declive *
**5**	5672	32.69	-57.03	-21.03	32	-58	-22	Posterior Lobe Declive *
**6**	5168	15.14	-78.8	-13.48	26	-86	-6	Inferior Occipital Gyrus Brodmann area 18
					-6	-74	-24	Posterior Lobe Pyramis *
					2	-70	-26	Posterior Lobe Tuber of Vermis *
					6	-72	-16	Posterior Lobe Declive *
**7**	2824	-26.43	-86.46	-4.77	-24	-90	-8	Inferior Occipital Gyrus Brodmann area 18
					-30	-86	2	Middle Occipital Gyrus Brodmann area 18
					-22	-78	-16	Posterior Lobe Declive *
**8**	384	-1.12	-57.01	-5.94	-2	-58	-6	Anterior Lobe Culmen *
**9**	264	55.34	-38.74	10.8	56	-40	12	Superior Temporal Gyrus Brodmann area 22

ALE MAPS WERE COMPUTED AT AN FDR-CORRECTED THRESHOLD OF P < 0.05; MINIMUM CLUSTER DIMENSION K>100MM3
